# Differential expression of histone H3 genes and selective association of the variant H3.7 with a specific sequence class in *Stylonychia* macronuclear development

**DOI:** 10.1186/1756-8935-7-4

**Published:** 2014-02-07

**Authors:** Sakeh Forcob, Aneta Bulic, Franziska Jönsson, Hans J Lipps, Jan Postberg

**Affiliations:** 1 Institute of Cell Biology, ZBAF, Centre for Biomedical Education and Research, Witten/Herdecke University, Witten, Germany; 2 Helios Medical Centre Wuppertal, Paediatrics Centre, Witten/Herdecke University, Wuppertal, Germany

**Keywords:** Histone variants, H3, Chromatin, Ciliates, Macronucleus, Micronucleus, Sexual reproduction, Piwi, ncRNA

## Abstract

**Background:**

Regulation of chromatin structure involves deposition of selective histone variants into nucleosome arrays. Numerous histone H3 variants become differentially expressed by individual nanochromosomes in the course of macronuclear differentiation in the spirotrichous ciliate *Stylonychia lemnae*. Their biological relevance remains to be elucidated.

**Results:**

We show that the differential assembly of H3 variants into chromatin is strongly correlated with the functional separation of chromatin structures in developing macronuclei during sexual reproduction in *Stylonychia*, thus probably determining the fate of specific sequences. Specific H3 variants approximately 15 kDa or 20 kDa in length are selectively targeted by post-translational modifications. We found that only the 15 kDa H3 variants including H3.3 and H3.5, accumulate in the early developing macronucleus, and these also occur in mature macronuclei. H3.7 is a 20 kDa variant that specifically becomes enriched in macronuclear anlagen during chromosome polytenization. H3.7, acetylated at lysine-32 (probably equivalent to lysine-36 of most H3 variants), is specifically associated with a sequence class that is retained in the mature macronucleus and therefore does not undergo developmental DNA elimination. H3.8 is another 20 kDa variant that is restricted to the micronucleus. H3.8 is selectively targeted by lysine methylation and by serine or threonine phosphorylation. Intriguingly, the expression and chromatin localization of the histone variant H3.3 was impaired during macronuclear differentiation after RNA interference knock-down of Piwi expression.

**Conclusions:**

Differential deposition of H3 variants into chromatin strongly correlates with the functional distinction of genomic sequence classes on the chromatin level, thus helping to determine the fate of specific DNA sequences during sexual reproduction in *Stylonychia*. Consequently, H3 variants are selectively targeted by post-translational modifications, possibly as a result of deviations within the recognition motifs, which allow binding of effector proteins. We propose that differential assembly of histone variants into chromatin of various nuclear types could contribute to nuclear identity, for example, during differential development of either new micronuclei or a macronuclear anlage from mitosis products of the zygote nucleus (synkaryon). The observation that the Piwi-non-coding RNA (ncRNA) pathway influences the expression and deposition of H3.3 in macronuclear anlagen indicates for the first time that selective histone variant assembly into chromatin might possibly depend on ncRNA.

## Background

Spatiotemporal coordination of gene expression, replication, repair, and developmental processes in eukaryotes are coordinated by the interplay of the genome and epigenetic signatures at various hierarchical levels, such as CpG signaling (that is, DNA cytosine methylation/hydroxymethylation) [1,2] and post-translational modification (PTM) contributing to chromatin structure regulation [3,4]. Evidence is accumulating that selective deposition of variant histones into nucleosomes and eventually chromatin via interactions with histone chaperones represents a further crucial level of chromatin structure regulation [5]. It is believed that the selective incorporation of histone variants into nucleosomal arrays leads to the establishment of cell type-specific ‘barcodes’, which can be transmitted to daughter nuclei in proliferating cells, thus contributing to the maintenance of cell type-specific gene expression patterns [6]. According to earlier studies, the histone variant H3.3 associates preferentially with euchromatin [7]. However, H3.3 is also suspected to fulfill more versatile functions during mammalian embryogenesis [8], in which murine paternal and maternal pronuclei adopt asymmetric H3.3 signatures. In detail, H3.3 associates preferentially with the paternal pronucleus, but is largely devoid of H3K4me3. Instead, H3.3 seems to be involved in the establishment of pericentric heterochromatin, which is required for proper chromosome segregation during the first mitosis, which follows pronuclei formation [9-11].

It has been argued that constitutively expressed variants may initially have evolved solely as replacement variants in non-cycling cells or between S-phases, when replication-dependent variants are absent. However, observations that diverse H3 variants evolved frequently but independently within related species in almost all eukaryotic supergroups oppose this view [12]. Instead, it is likely that that multiple H3 variants evolved to fulfill diverse functions in the cell cycle and development of various eukaryotic lineages, despite their extremely high degree of protein sequence conservation.

In addition to being found in metazoa, histone H3 variants are commonly found in single-celled ciliated protozoa, such as *Tetrahymena*[13] or *Euplotes*[14]. Even within the ciliophora phylum, *Stylonychia* occupies an exceptional position. Recently, we characterized full-length macronuclear genomic sequences encoding eight histone H3 variants [12], which had been fragmentarily identified more than a decade ago [15]. To date, this is the highest number of H3 variants found in a single species, except for humans. Thus, this ciliate species could be an attractive model for the study of the spatiotemporally coordinated expression of histone variants, their assembly into chromatin, and their biological relevance.

Ciliates are characterized by nuclear dualisms, whereby each cell contains two different nuclear types: somatic macronuclei and germline micronuclei (see Additional file 1: Figure S1A, step 1). Transcripts required for vegetative growth are synthesized in the macronucleus, whereas the transcriptionally inert micronuclei consist of condensed chromatin [16]. The macronuclear DNA of the stichotrichous ciliate species *Stylonychia lemnae* is organized in short molecules, known as nanochromosomes, ranging in size from 0.4 to 75 kb. Each of these nanochromosomes usually contains one open reading frame and all the sequences required for expression and replication. Sexual reproduction (conjugation) leads to the differentiation of a new macronucleus from a micronuclear derivative, while the parental macronucleus becomes degraded (see Additional file 1: Figure S1A, steps 2 to 6). The latter starts at the onset of conjugation and at the same time, micronucleus meiosis takes place (see Additional file 1: Figure S1A, step 2). Subsequently, haploid migratory micronuclei become exchanged between conjugation partners (see Additional file 1: Figure S1A, step 3, A_inset_). By fusion, these migratory nuclei build a synkaryon with their stationary counterparts, which is followed by mitosis. One of the resulting products of this mitosis will build a new micronucleus, whereas the other product (anlage) will develop into a new macronucleus (see Additional file 1: Figure S1A, step 4). In *Stylonychia*, a first phase of sequential DNA replication events leads to polytene chromosomes formation in the macronuclear anlagen followed by a programmed loss of micronucleus-specific DNA sequences (Additional file 1: Figure S1A, steps 5–6). Thus the DNA content in the developing macronucleus changes dramatically over time (Additional file 1: Figure S1B). Micronucleus-specific DNA largely consists of ‘bulk’ repetitive and transposon-like elements, and of internal eliminated sequences (IES), which interrupt macronucleus-destined sequences (MDSs) in a large portion of scrambled genes, whose modules have to be reordered during macronuclear development [17]. During these processes, dramatic DNA reorganization and elimination processes take place. Over 90% of micronuclear sequences become organized into condensed chromatin domains, which are eventually excised from the genome [18,19]. Macronucleus maturation is accompanied by a second phase of sequential DNA replication events, leading to the final copy numbers of nanochromosomes. Conjugation is associated with a short-term boost of differential gene expression, and many of these expressed genes are suspected to be involved in the regulation of programmed genome reorganization. Among these genes are histone variants and a Piwi family protein [20,21]. Furthermore, small non-coding RNAs (ncRNAs) accumulate, which may result from short-term transcription of the micronuclear genome, as reported for *Tetrahymena*[22]. By contrast, recent studies suggest a parental macronuclear origin of ncRNA in *Oxytricha*, a species closely related to *Stylonychia*[23,24]. For *Stylonychia*, the nuclear localization of ncRNA synthesis remains unsolved, and some earlier observations support a possible micronuclear origin [16,25]. However, it is believed that these ncRNAs eventually interact with Piwi, and undergo a selection process via comparison with the parental macronuclear genome, resulting in a subfraction of ncRNAs homologous to specific sequences. Finally, Piwi-bound ncRNAs target homologous sequences in the developing macronucleus, which are then converted into discrete chromatin structures [26].

Here we provide detailed insight into differential H3 gene expression patterns and the accumulation of three H3 variant proteins during macronuclear differentiation in *Stylonychia*. We show that some H3 variants are spatiotemporally regulated, and possess specific PTM signatures. In polytene anlagen, acetylated H3.7 is associated with specific sequence classes. Perturbation of the Piwi-ncRNA pathway leads to impaired *HIS33* gene expression, and entails decreased deposition of H3.3 protein levels in anlagen chromatin, suggesting a link between the mechanisms responsible for RNA-directed chromatin-reorganization and the expression of some H3 variants.

## Results

### Eight non-redundant histone H3 variants are expressed from nine nanochromosomes in the life cycle of *Stylonychia*

To obtain the full-length nanochromosomes encoded in the macronucleus genome of *Stylonychia*, we applied telomere-suppression PCR [27], confirming the presence of nine discrete nanochromosomes (see Additional file 2: Figure S2A). We had earlier included the protein sequences of these nanochromosomes in a study about the evolutionary history of histone H3 in eukaryotes [12]. Two of the nine nanochromosomes, *HIS32A* and *HIS32B*, encoded almost identical proteins. The single difference was H3.2aS29/H3.2bL29 (see Additional file 3: Figure S3). Another almost identical histone H3 variant was H3.1, encoded by *HIS31*, which had H3.1S29/C122 instead of A112 in H3.2a/b. We previously proposed that all *Stylonychia* H3 variants had evolved from a H3.3-like ancestor [12]. H3.3 and H3.5, encoded by *HIS33* and *HIS35*, were most reminiscent of H3.3 in *Hydra* or nuclearids, which resembled the putative ancestral protoH3 [12]. Further, both H3.4 and H3.6, encoded by *HIS34* or *HIS36*, respectively, were closely related to H3.3. The only variant containing a GT-AG-type intron was *HIS33*. In contrast to these variants, whose coding sequence (CDS) size was between 411 and 417 nucleotides (nt) with predicted molecular weights of 15.25 to 15.75 kDa (Table 1), two more deviant variants had evolved. H3.7 (gene *HIS37*) had a predicted size of 20.01 kDa and consisted of 543 nt in the coding region. Most of the deviations in H3.7 occurred within the N-terminus. Of similar size was H3.8 (predicted size 20.48 kDa). Deviations in H3.8 were also found in the N-terminus, and additional residues were attached to the C-terminus. BLAST searches using the *Stylonychia* macronuclear genome draft database (http://stylo.ciliate.org/) provided no evidence for further H3 variants.

**Table 1:** **Features of *Stylonychia *H3 variants**

**Gene**	**5′-telomere**	**5′-sub-telomere**	**CDS**	**3′-sub-telomere**	**3′-telomere**	**Total**	**Intron**	**Protein**	**MW**	**Nuclear type**	**PTM**	**Sequence class**	**Piwi-dependent**
*HIS31*	20	728	417	283	36	1484	NA	H3.1	15.59				No
*HIS32A*	20	730	417	313	36	1516	NA	H3.2a	15.56				No
*HIS32B*	20	727	417	313	36	1513	NA	H3.2b	15.59				No
*HIS33*	20	198	417	154	36	893	68: 399 to 466	H3.3	15.75	M, a1 to a3, e			Yes
*HIS34*	20	169	411	240	36	876	NA	H3.4	15.69				Uncertain
*HIS35*	20	233	417	255	36	961	NA	H3.5	15.25	M, a1 to a3, e			Uncertain
*HIS36*	20	231	414	467	36	1168	NA	H3.6	15.56				ND
*HIS37*	20	429	543	255	36	1283	NA	H3.7	20.01	a3, e	H3.7K3me3 (H3K4me3); H3.7K105ac (GVKacKPHR)	MDS loci	No
*HIS38*	20	97	540	327	36	1020	NA	H3.8	20.48	m	H3.8K26me3 or H3.8K32me3 (H3K27me3); H3.8S27ph or H3.8T33ph (H3S28ph)		No

*Abbreviations*: *a1 to a3* anlagen during polytenization, *e* anlagen during DNA elimination, *CDS* coding sequence, *M* macronucleus, *m* micronucleus, *MDS* Macronucleus-destined sequence, *MW* molecular weight, *NA* not applicable, *ND* not done, *PTM* post-translational modification.

**Figure 1:**
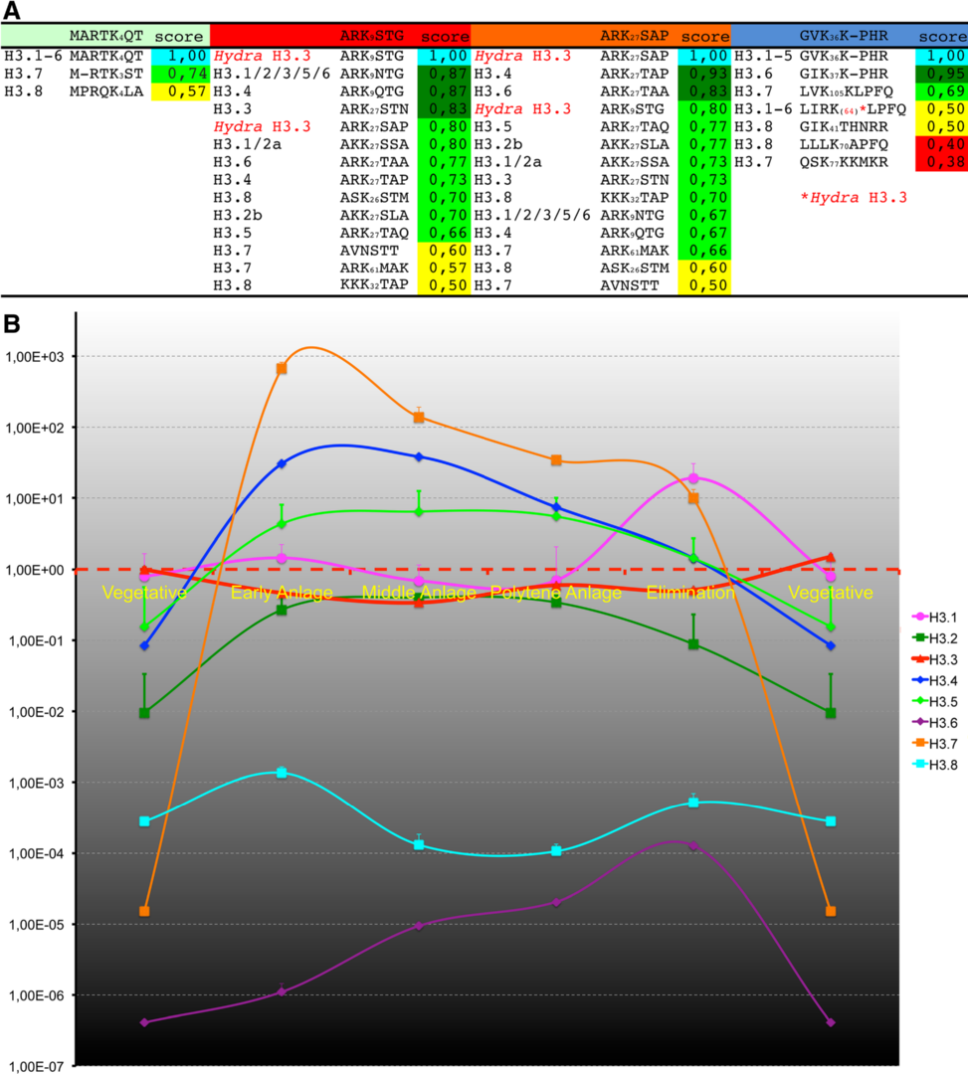
**Conservation of post-translational modification (PTM) targets in *Stylonychia *H3 variants and the accumulation of H3 variant mRNAs during macronuclear differentiation. (A)** A similarity matrix of sequence motifs adjacent to well-characterized PTM target sites exhibited similarities and differences between several H3 variants. A match score was calculated between two aligned amino acids using an amino acid class hierarchy diagram [28]. **(B)** The relative abundance of several H3 variant mRNAs changed over time. Accumulation of *Stylonychia* H3 variants mRNA during macronuclear development was assessed by quantitative PCR (qPCR). Prior to cDNA synthesis, RNA was isolated from synchronized cells at several developmental stages, which corresponded to the time line (*x*-axis) as follows: 1) during vegetative growth phase; 2) from cells after conjugation, when an early anlagen nucleus was visible; 3) from cells with polytene chromosome anlagen nuclei prior to bulk DNA elimination; 4) from cells containing polytene anlagen nuclei at the onset of bulk DNA elimination; and 5) from cells within the DNA-poor anlagen stage. Values represent mean and standard deviation (SD), and only the upper error bar is shown. All values were normalized to H3.3 mean mRNA levels during vegetative growth. Extensive enrichment of H3.7 and H3.4 mRNAs was observed during the first round of DNA amplification leading to polytene chromosomes. Intermediate levels of H3.5 mRNA were measured during chromosome polytenization, whereas H3.1 mRNAs accumulated during the second round of DNA amplification, leading to the final copy numbers of mature nanochromosomes.

Strikingly, the most prominent differences between these variants occurred within sequence motifs known to be targets of chromatin-modifying enzymes. These motifs included all the above residues adjacent to H3K27, and also the similar motif adjacent to H3K9 (see Additional file 3: Figure S3; referring to numbering in *Hydra* histone H3). Unless otherwise indicated, we ignore the correct numbering of *Stylonychia* H3 variant residues, which is often deviant, to ease comparability between homologous motifs. A complete similarity matrix of these homologous motifs with correct numbering is provided in Figure 1A. Lysine-27 was conserved in all histone H3 variants, and lysine-9 in almost all of these variants, except H3.7. At least two main groups may be relevant, which contained either AKK_27_S (H3.1, H3.2) or ARK_27_S/T. Notably, serine-10, which is usually conserved in animal H3.3, was not found in most *Stylonychia* H3 variants, except in H3.8 within the ASK_26_S motif. By contrast, H3K27 was accompanied by serine or threonine in almost all variants, except H3.7 (ARK_61_M).

The motif adjacent to H3K36 (GVK_36_K-PHR) was identical to animal H3 in H3.1 to H3.5 and almost identical in H3.6, but it deviated at homologous loci of H3.7 and H3.8. Interestingly, a very similar motif had evolved in H3.7 (LVK_105_KLPFQ), directly before the N-terminal end of the α1 helix adjacent to the histone fold domain. The H3K4 motif (ARTK_4_QT) did not differ from animals in H3.1 to H3.6, except in H3.7 and H3.8.

The transfer and deposition of histone variants into chromatin is mediated via their association with specific histone chaperones. For example, Asf1 is involved in the transfer of H3-H4 dimers, and acts as a donor for the variant specific chaperone complexes CAF-1 (replication-dependent; specificity for H3.1-H4 dimers) or HIRA (replication-independent; specificity for H3.3-H4 dimers) [29]. Therefore, another region of interest was the chaperone recognition domain, which stretches over loop L1 and the α2 helix in the histone fold domain (see Additional file 3: Figure S3). A remarkable number of deviating residues were found in the chaperone recognition sites in *Stylonychia* H3 variants, and these domains were identical in H3.1 and H3.2. The chaperone recognition domains in H3.3 and H3.5 differed in only one residue (H3.3 L102/H3.5 M102), but both were different from H3.1/H3.2. All other variants exhibited more differences, as confirmed by analyses of the phylogenetic distances (see Additional file 3: Figure S3B).

Next, we induced sexual reproduction of different *Stylonychia* mating types. The discrete morphological differences of the nuclei allowed us to evaluate the synchronicity of the cells, which was over 90%. Cells were harvested at various developmental stages, including vegetative macronuclei, macronuclear anlagen during polytenization (a1 to a3), and anlagen during bulk DNA elimination towards the DNA-poor stage (see Additional file 1: Figure S1). RNA was then isolated and reversely transcribed to cDNA. We used quantitative real-time PCR (qPCR) to monitor the accumulation of each histone H3 variant mRNA at all time points with reference to their levels in vegetative cells (Figure 1B). During macronuclear development, extensive enrichment of some of the H3 variant mRNAs was observed either during the first round of replication, which leads to chromosome polytenization (H3.7, H3.4, H3.5), or during the second round of nanochromosome replication, in the course of macronucleus maturation (H3.1). Therefore, we consider H3.1, H3.4, H3.5 and H3.7 to be replication-dependent variants. All other variants were less subject to variation, and appeared to be permanently expressed on a lower level over the *Stylonychia* life cycle.

### H3 variants exhibit differential spatiotemporal localization during macronuclear development

**Figure 2:**
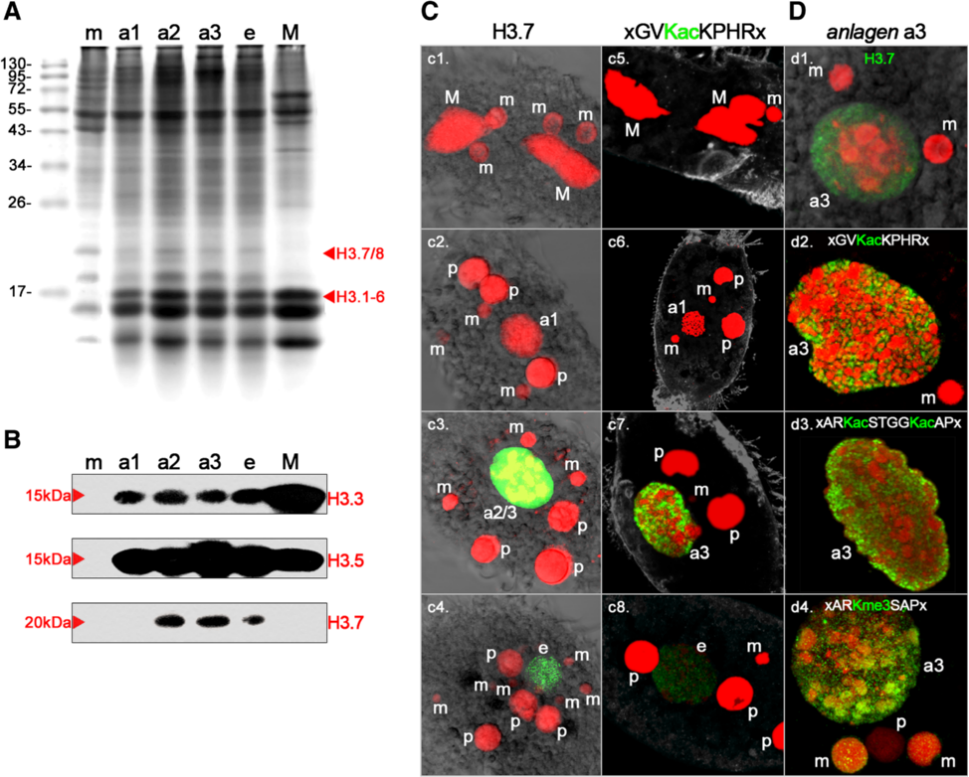
**Spatiotemporal occurrence of H3 variant proteins, nuclear localization of H3.7 and selected post-translational modifications (PTMs). (A)** Nuclear proteins were isolated from micronuclei (m), early anlagen (a1) with visible chromatin decondensation, mid anlagen (a2) with polytene chromosomes prior to bulk DNA elimination, late anlagen (a3) at the onset of DNA elimination, DNA-poor anlagen (e) during extensive DNA elimination, and macronuclei (M). The proteins were separated by SDS-PAGE and stained with Coomassie Brilliant Blue. Red arrows indicate bands corresponding to 20 kDa (H3.7, H3.8) and 15 kDa H3 variants (H3.1 to H3.6). **(B)** Western blot analyses were performed using the same samples as described in **(A)** for SDS-PAGE. Antibodies targeted to H3.3, H3.5, or H3.7 were used for detection. **(C) ***In situ* antibody staining using primary antibodies targeted to histone H3.7 (c1 to c4) or H3K36ac (c5 to c8) (green) and DNA counterstaining (red). The cellular shape was visualized in c5 to c8 using an α-tubulin-antibody (grey). All images are confocal image stack projections of 5 to 10 images from the middle of the stacks. Abbreviations: m, micronuclei; M, macronuclei; a1 to a3, macronuclear anlagen during the first round of DNA amplification (compare above); e, macronuclear anlagen towards the DNA-poor stage; p, parental/old macronuclei. **(D)**: Details of macronuclear anlagen (a3) using antibodies targeted to H3.7 (d1), H3K36ac (d2), H3K9ac/k14ac (d3) or H3K27me3 (d4). The lettering and color scheme is as described in **(C)**.

Proteins purified from micronuclei, vegetative macronuclei, and macronuclear anlagen at successive developmental stages were separated by SDS-PAGE, and Coomassie staining was performed (Figure 2A). In micronuclear (m) protein extracts, prominent H2A/H2B and H4 bands could be observed, but there was no H3 band with a size of about 15 kDa. Instead, a 20 kDa band was visible, representing ‘protein X’, which has been proposed as an H3 replacement variant [30]. In extracts from macronuclear anlagen during polytenization (a1 to a3) and during DNA elimination (e) as well as in vegetative macronuclei (M), a full set of histone bands representing 15 kDa H3 variants, H2A/H2B, and H4 were evident. Moreover, a 20 kDa band emerged in early anlagen (a1), was prominent in advanced polytenization stages (a2 and a3), and decreased in abundance during the DNA elimination (e) stage. Another 16–18 kDa band not present in macronuclei was seen in micronuclei and anlagen, but none of the H3 variants identified to date corresponds to this protein weight.

Differences in some of the H3 variants seemed to be promising epitopes for antibody production. Thus, we raised polyclonal antibodies (pAbs) targeted against three histone H3 variant peptides: H3.3 (guinea pig), H3.5 (rabbit), and H3.7 (rat). We then performed Western blot analyses using the same developmental stage samples used for SDS-PAGE and blotting as described above. These experiments confirmed that the accumulation of H3 variant proteins correlated with the enrichment of mRNAs (Figure 2B). In detail, H3.3 was present as a 15 kDa band in macronuclei (M), and in macronuclear anlagen (a1 to a3, e), but not in micronuclei. The band intensity appeared to be directly correlated with the H3 band intensity in the Coomassie-stained gel (Figure 2A). Similarly, H3.5 (15 kDa) was not found in the micronucleus (m), but was found in all other developmental stages and the macronucleus. The highest band intensity was seen in anlagen during the highest degree of polytenization, which was in agreement with the accumulation of H3.5 mRNA (Figure 1B). H3.7 emerged as a 20 kDa band in mid anlagen during polytenization (a2), in a slightly deferred manner compared with H3.7 mRNA enrichment. H3.7 was present in anlagen with the highest degree of polytenization (a3) and during DNA elimination (e), but it could not be detected in micronuclei (m) or macronuclei (M). To study the spatiotemporal localization of H3.7 in detail, we performed immunofluorescence microscopy using anti-H3.7 pAbs (Figure 2C). Unfortunately, the antibodies targeted to H3.3 and H3.5 turned out to be unsuitable for *in situ* antibody staining.

H3.7 was not detected in micronuclei (m) or macronuclei (M) in vegetative cells (Figure 2C1). It was also not found in early developing macronuclear anlagen (a1), micronuclei (m) or fragments of the parental macronucleus (p) or in cells that had separated after conjugation (Figure 2C2). Strikingly, and in agreement with *HIS37* mRNA accumulation as well as Western blot analyses, H3.7 was strongly enriched in cells containing macronuclear anlagen with a high degree of chromosome polyteny (a2/a3). H3.7 was sharply restricted to these nuclei, and did not occur in micronuclei (m) or parental macronuclear fragments (p) (Figure 2C3). Similarly, H3.7 could still be detected in anlagen during programmed DNA elimination (e), but not in other nuclear types (Figure 2C4). To uncover the potential relevance of H3.7 for programmed chromatin reorganization, we silenced its expression using RNA interference (RNAi). We could not observe an effect of this treatment on vegetative *Stylonychia*. Upon mixing of different mating types, only a few cells underwent conjugation. However, we could not observe developmental progression, and usually the cells died within a few hours.

With respect to earlier studies of numerous spatiotemporal histone H3 PTM patterns in nuclei during sexual reproduction in *Stylonychia*[16], we noticed that the H3.7 signature was reminiscent of the signals obtained when anti-H3K36ac pAbs were used for immunofluorescence staining, which were raised using a peptide containing the motif GVKacKPHR (Figure 2C5-8). Of all the histone acetylation markers examined so far during macronuclear development in *Stylonychia*, the H3K36ac signature is unique insofar as the detected PTM is restricted to macronuclear anlagen. By contrast, earlier studies have shown that other acetylated H3 residues, such as H3K9ac or H3K14ac, also occur in vegetative macronuclei and parental macronuclear fragments, and already accumulate in very early macronuclear anlagen stages [16]. When we examined single confocal optical sections of highly polytene macronuclear anlagen (a3) in detail, the signatures of H3.7 were reminiscent of those seen when antibodies targeting H3K36ac or H3K9ac/K14ac (motif contained in immunizing peptide ARKacSTGGKacAP) were used (Figure 2D1-3). Neither H3.7 nor any of the PTMs could be detected in micronuclei (m) or parental macronuclear fragments (p). Instead, signals corresponding to H3.7, H3K36ac, or H3K9ac/K14ac were strongly enriched in discrete domains in macronuclear anlagen, which exhibited rather weak DNA staining, whereas these signals were totally absent from the so-called heterochromatic blocks, which exhibited intense DNA staining. A spatiotemporal signature similar to H3K36ac was also observed when we used antibodies targeted to H3K27me3, which were raised using a peptide containing the motif ARKme3SAP. In addtion, H3K27me3 signals emerged in later macronuclear anlagen stages (a3), persisted in the DNA-poor anlagen (e), and vanished during the course of macronuclear maturation [16]. When these results were compared in detail, it became obvious that H3K27me3, in contrast to H3K36ac or H3K9ac/K14ac, was also enriched within the heterochromatic blocks (Figure 2D4).

### H3.7 becomes specifically acetylated during development

**Figure 3:**
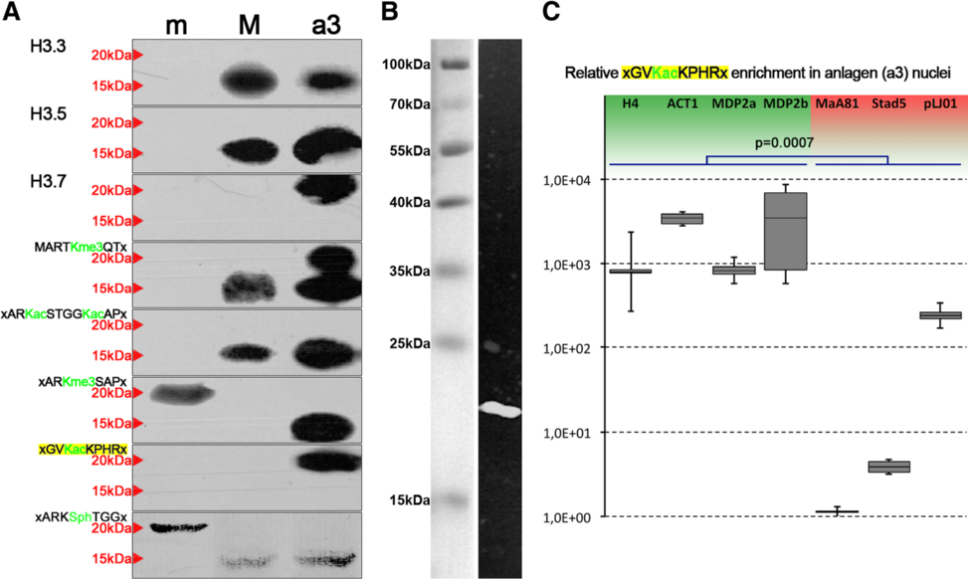
**Nuclear localization of H3 variants and post-translational modifications (PTMs) and results of chromatin immunoprecipitatino (ChIP) analyses on chromatin purified from polytene anlagen (a3) using a pAb targeted to H3K36ac. (A)** Western blot analyses of nuclear proteins isolated from micronuclei (m), macronuclei (M) and polytene anlagen (a3). Antibodies used were targeted against H3.3, H3.5, H3.7, H3K4me3, H3K9ac/K14ac, H3K27me3, H3K36ac, and H3S10ph. Red arrows indicate bands corresponding to 20 kDa or 15 kDa H3 variants. **(B)** Results of Western blot analyses post-immunoprecipitation. Antibodies targeted to H3K36ac were used for ChIP on polytene anlagen (a3) chromatin. Immunocomplexes were separated by SDS-PAGE and analyzed by Western blots using anti-H3.7 pAbs for detection. Left lane: protein size standard visualized by direct light photography; right lane: fluorescence light detection of a 20 kDa protein band. **(C)** Enrichment of several model sequences representing MDSs (H4, ACT1, two loci within MDP2) or micronucleus-specific sequences (MaA81, Stad5, pLJ01) was studied. Box plots exhibit median, interquartile range, and minimum and maximum values.

To obtain proof that H3.7 is targeted by the specific acetylation that was detected using the H3K36ac antibody (GVKacKPHR), we performed Western blot analyses using the same samples as described above, in combination with PTM-specific antibodies. Even though there is a perfectly matching motif in the 15 kDa H3 variants H3.1-5 (Figure 1A, score = 1.0) this antibody did not react with these variants in Western analyses, either in the same nuclei or in micronuclei (m) or macronuclei (M), showing that these H3 variants were not acetylated at this site. Intriguingly, although H3.7 possesses a weaker matching motif (Figure 1A, score = 0.69) the anti-H3K36ac antibodies reacted with a 20 kDa band in polytene anlagen (a3), (Figure 3A, bottom), but not in micronuclei (m) or macronuclei (M). To test whether the anti-H3K36ac antibody reacted with H3.7, we used SDS-PAGE to separate chromatin proteins pulled down with this antibody, which was followed by electrotransfer onto a PVDF membrane and immunodetection using rat anti-H3.7 polyclonal antibodies in combination with goat anti-rat-biotin IgG (Abcam, Cambridge, UK) and Qdot 625 streptavidin conjugate (Molecular Probes, Eugene, Oregon, USA). We detected a band of approximately 20 kDa, showing that H3.7 was present in the immunocomplex pulled down with the anti-H3K36ac antibody (Figure 3B).

Similarly, H3.7 also reacted with anti-H3K4me3 antibodies, and these antibodies also reacted with 15 kDa H3 variants in anlagen (a3) and macronuclei (M). To complement previous data, which showed spatiotemporal histone H3 PTM pattern during macronuclear development [16], and to assign specific PTMs to particular histone H3 variants, we investigated whether antibodies targeted to other PTMs would also react with the 20 kDa or 15 kDa variants in the different nuclear types (Figure 3A). Antibodies targeted to H3K4me3 (motif MARTKme3QT) reacted with the 15 kDa H3 variants in macronuclei and anlagen (a3), but not with any micronuclear (m) variant. Micronuclei were also devoid of H3K9ac/K14ac (motif ARKacSTGGKacAP). This modification was enriched in macronuclei (a3) and anlagen (a3), and antibodies reacted exclusively with a 15 kDa band. Antibodies targeted to H3K27me3 exhibited differential reactivity. A reaction with a 20 kDa H3 variant was seen in micronuclei (m), whereas the antibodies reacted with a 15 kDa band in macronuclear anlagen (a3). There was no reaction with any band observed in macronuclei (M).

### Acetylated H3.7 is enriched in macronucleus-destined sequences in polytene chromosomes containing anlagen

We were particularly interested in whether sequences belonging to the classes mentioned above (that is, macronucleus-destined sequences, MDSs, or bulk DNA sequences removed during macronuclear development) were preferentially associated with nucleosomes that contained specific histone H3 variants. Unfortunately, none of the raised antibodies was suitable for chromatin immunoprecipitation (ChIP). Instead, we decided to make use of an indirect strategy to achieve enrichment of DNA sequences associated with H3.7-containing nucleosomes. Because only H3.7 was observed being targeted by H3K36ac-like PTM, and this PTM took place during the macronuclear developmental stage of interest (a3), we used the anti-H3K36ac pAb for ChIP. We then performed qPCR to investigate whether sequences corresponding to MDSs (micronuclear histone *h4* gene, *actin I*, or two loci within *mdp2*) or to micronucleus-specific sequences eliminated during macronuclear differentiation (MaA81, Stad5, pLJ01) were enriched in the precipitated chromatin. We found that enrichment of sequences belonging to the MDS class significantly exceeded the amount of micronucleus-specific sequences (Figure 3C).

### Knock-down of Piwi results in impaired *HIS33* gene expression and deposition of H3.3 into anlagen chromatin

Data obtained in this study strongly suggest that at least some of the histone H3 variants in *Stylonychia* could be important determinants in the control of programmed chromatin reorganization during macronuclear differentiation. It is believed that these processes are driven by small ncRNAs, which interact with the Argonaute protein family member Piwi. Piwi appears to be involved in ncRNA turnover and eventually in the determination of DNA sequences, which become subject to programmed chromatin reorganization. Similarly to Otiwi1 in *Oxytricha*, which has been described very recently [23], a Piwi homolog was found in *Stylonychia*, and is the most abundant protein that is differentially expressed at the onset of macronuclear development [20,21]. Its knock-down by RNAi led to a loss of Piwi protein below the detection sensitivity of Western blot analyses [16] and to arrest in macronuclear development [25]. The differential spatiotemporal distribution of this protein suggests that Piwi is involved in trans-nuclear cross-talk [16].

**Figure 4:**
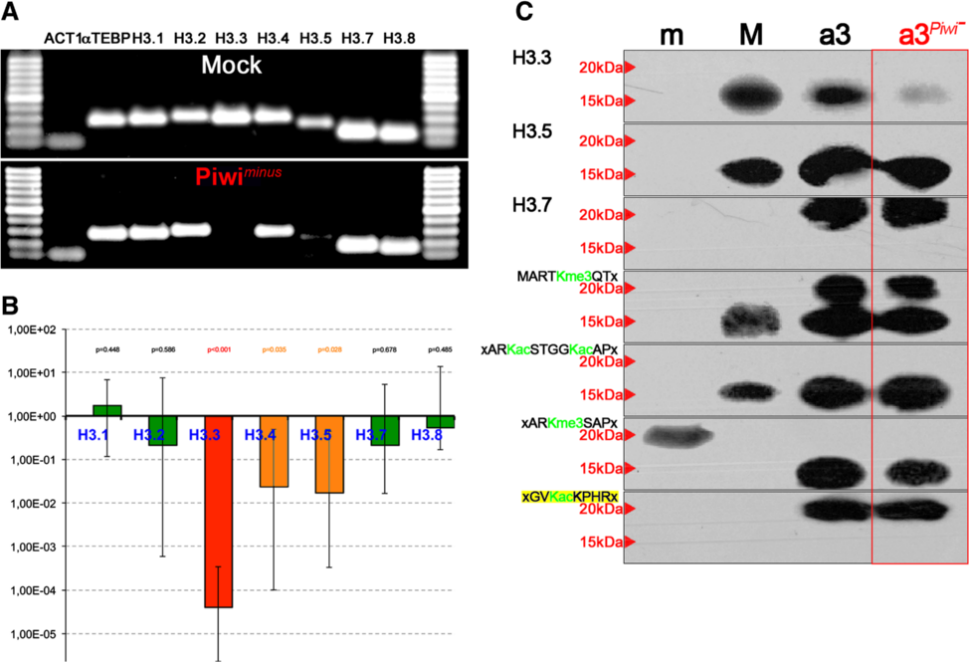
**Expression of *HIS33 *and deposition of H3.3 into polytene anlagen chromatin (a3) depends on the Piwi-non-coding RNA (ncRNA) pathway.** The effects of Piwi knock-down on the expression of H3 variant mRNAs were studied by semiquantitative PCR **(A)** and quantitative PCR **(B)**. **(C)** Expansion of Figure 3A: Western blot analyses of nuclear proteins isolated from micronuclei (m), macronuclei (M) and polytene anlagen (a3) as well from anlagen (a3) after Piwi-minus RNAi treatment. Antibodies used were targeted against H3.3, H3.5, H3.7, H3K4me3, H3K9ac/K14ac, H3K27me3, H3K36ac, and H3S10ph. Red arrows indicate bands corresponding to 20 kDa or 15 kDa H3 variants.

Consequently, we decided to make a simple attempt to investigate the potential relevance of the Piwi-ncRNA pathway for the deposition of histone H3 variants involved in macronuclear development. Therefore, we targeted Piwi mRNAs by RNAi in preliminary experiments. We studied the effects of Piwi RNAi (Piwi-minus) on the levels of histone variant mRNAs, using semiquantitative PCR, agarose gel electrophoresis (Figure 4A) and qPCR (Figure 4B), and studied the effects of Piwi-minus on the levels of protein using Western analyses on chromatin obtained from macronuclear anlagen (a3) (Figure 3C). Further, we analyzed whether RNAi influences several H3 PTMs. Interestingly, quantification of histone H3 variant mRNAs in three technical replicates of Piwi-minus knock-downs revealed that the histone H3 variant H3.3 became significantly silenced (*p* < 0.01) (Figure 4B). This finding was supported by endpoint PCR and subsequent agarose gel electrophoresis (Figure 4A); no H3.3 band could be observed after Piwi-minus knock-down. By contrast, expression of H3.1, H3.2, H3.7, and H3.8 was not changed, as shown by qPCR and endpoint PCR. It further appeared that similarly to H3.3, H3.4 and H3.5 could also be down-regulated by Piwi-minus knock-down, but the statistical support was weaker than for H3.3. However, in endpoint PCR analyses using agarose gel separation, we observed a very weak band of H3.5 in response to RNAi treatment, whereas no intensity change was seen for H3.4 with respect to mock controls. The variant H3.6 could not be observed in either Piwi-minus knock-downs or mock controls in this experiment. Strikingly, Western blot analyses confirmed that the H3.3 protein disappeared from macronuclear anlagen chromatin in Piwi-minus knock-down experiments (Figure 4C). Our perception that H3K27me3 was also very slightly down-regulated by Piwi-minus knock-downs is tentative, but it cannot be excluded. However, no evidence was seen that Piwi-minus knock-down impaired the accumulation of H3.5 and H3.7 or of specific PTMs (H3K4me3, H3K9ac/K14ac, H3K27me3, or H3K36ac).

## Discussion

### Sequence deviations in the recognition motifs of chromatin-modifying proteins suggest selective indexing of H3 variants by PTMs

Expanding our prior work, we undertook a detailed characterization of the numerous histone H3 encoding nanochromosomes as well as the encoded H3 variant proteins. We hypothesize that all or many of the numerous *Stylonychia* H3 variants with their discrete protein sequence differences serve as specific substrates for chromatin-regulating mechanisms. Interestingly, a hot spot of sequence variation was the motif adjacent to lysine-27 (see Additional file 3: Figure S3). The relevance of these motif variations should be discussed in close context with the very similar motif adjacent to lysine-9. When trimethylated at lysines-9/27, these motifs may act as binding modules for heterchromatin protein 1 (HP1)-like chromodomain proteins, whose protein family members were shown to participate in opposing functions such as transcriptional repression and activation. HP1 family members play key roles in the formation of repressed chromatin states through binding of H3K9me3 or H3K27me3, although their functions do not seem to be restricted to gene silencing. Such interactions have been described in various eukaryotes, including mammals, *Drosophila* and *Caenorhabditis elegans* (H3K9me3/HP1; H3K27me3/polycomb protein Pc), fission yeast (H3K9me3/Swi6), and ciliates such as *Tetrahymena* or *Stylonychia* (H3K9me3 and/or H3K27me3/Pdd1p). The similarity score for both sites within *Hydra* H3 is about 0.80, and the score range between *Hydra* and the more conserved *Stylonychia* H3 variants was 0.66 to 0.93 (Figure 1A). Even in H3.7 and H3.8, less conserved remnants of these motifs were found (scores 0.50 to 0.60). The observed deviations suggest that the affinity of effector proteins (‘readers’ and ‘writers’ of PTM signatures) may differ between specific H3 variants. Thus, *Stylonychia* H3 variant sequences could predetermine a range of biological functions by restricting the accessibility of modified or unmodified sites for chromatin-modifying proteins to an extent that is to date not known in other eukaryotes.

We discussed in a previous study whether deviant H3 variants, such as H3.7 or H3.8, could represent CENP-A homologs [12]. Briefly, we argued that their phylogenetic grouping between H3 and CENP-A variants could be due to long-branch attraction. Our current results show that H3.7 was associated with MDSs, and that this variant did not occur in micronuclei, to which conventional centromere-containing chromosomes are restricted in *Stylonychia*. We therefore can now rule out the possibility that H3.7 is a functional CENP-A homolog. H3.8 was the only H3 variant detected in micronuclei. It seems improbable that H3.8 is restricted to centromeres of micronuclear chromosomes. However, we cannot exclude the possibility that whole micronuclear chromosomes adopt a centromere-like chromatin structure.

### Micronuclear H3.8 is targeted by specific PTMs and becomes replaced during anlagen formation

As described previously [16], micronuclei reacted with antibodies targeted either to H3S10ph/S28ph or H327me3. In the current study, we made use of this observation to provide evidence that the 20 kDa variant H3.8 is the putative histone variant ‘protein X’ [30]. As shown by the Western blotting and microscopy results, the second 20 kDa variant H3.7 was restricted to the later macronuclear anlagen (a2 to a3, e). Therefore, the exclusive micronuclear 20 kDa variant must be H3.8. Antibodies targeted to H3K27me3 or H3S10ph/S28ph, respectively, reacted with this 20 kDa H3 variant in micronuclei (Figure 3A). KKK32TAP (score 0.70) or ASK26STM (score 0.60) are the most convincing target sites for the described lysine trimethylation, and furthermore, the adjacent threonine or serine are the most convincing phosphorylation targets. All micronuclei exhibited H3S10ph/S28ph in conjugating cells, when anti-H3S10ph/S28ph pAbs were used for immunofluorescence. Interestingly, it has been shown after separation of conjugating cells, those signals were lost [16]. Prior to exconjugant separation in *Stylonychia*, two DNA replication events take place (before postmeiotic mitosis and before synkaryon division), each possibly being a time point for extensive nucleosome assembly and replacement of H3.8 by new H3 variants (see Additional file 1: Figure S1, steps 3 and 4).

### Conservative H3 variant deposition during DNA replication could contribute to macronuclear anlagen identity

At the onset of sexual reproduction, haploid migratory micronuclei become exchanged between conjugation partners. These nuclei fuse to build a synkaryon with their haploid micronuclear counterparts, followed by mitosis. One of the resulting mitosis products will build a new H3.8-containing micronucleus, whereas the other product will develop into a new macronucleus. Importantly, we found extensive *de novo* enrichment of 15 kDa H3 variants in early macronuclear anlagen (a1), at which time a 20 kDa histone fraction was still visible (Figure 2A). Therefore, it seems probable that there is extensive exchange of H3.8 for a 15 kDa variant during the DNA replication event, which follows the synkaryon formation. The persistence of H3S28ph signals in micronuclei during conjugation gives support to this proposed timing. A conservative model of nucleosome deposition to the daughter strands of newly replicated DNA therefore seems conceivable, in which octamers containg the 15 kDa H3 variant become selectively assembled with the DNA of the strand that gives rise to the macronuclear anlagen genome (see Additional file 1: Figure S1A, step 4). With respect to these findings, we speculate that nuclear identity determination could involve such a mechanism, by which chromatin of the new micronucleus eventually contains H3.8, and the chromatin of the prospective macronucleus contains mainly 15 kDa H3 variants, such as H3.3 and H3.5. We have described above that both variants occur in the early macronuclear anlagen.

### H3.7 in its acetylated form is associated with a specific class of sequences in anlagen during macronuclear differentiation

Expression of H3.7 takes place early during macronuclear development, and the H3.7 protein accumulates exclusively in macronuclear anlagen (a2, a3) during micronuclear chromosome polytenization, and is present until the end of programmed DNA elimination in the DNA-poor stage (e). It seems obvious that H3.7 is involved in chromatin regulation processes in anlagen nuclei. Our data suggest that H3.7 alone reacted with anti-H3K36ac pAbs, indicating a unique PTM targeted to H3.7. In light optical sections, it became evident that both acetylated H3.7 and acetylated 15 kDa H3 variants exhibited similar nuclear distribution, overlapping with domains of decondensed chromatin. These observations pinpoint to a contribution of H3.7 to the establishment of a permissive chromatin structure. Indeed, H3.7 was associated with MDSs, a finding that was reminiscent of acetylated 15 kDa variants [16], but with respect to the discrete differences in their spatiotemporal accumulation, it possibly indicates non-redundant functional relevance.

All H3 acetylation markers were omitted from heterochromatic blocks or H3K27me3 signals. Moreover, in Western blot analyses, H3.7 did not react with anti-H3K27me3 pAbs, the primary hallmark for heterochromatic blocks. Although it thus seems improbable that H3.7 carrying an H3K27me3-like PTM was associated with micronucleus-specific sequences, we cannot exclude an association of non-acetylated H3.7 with such sequences. However, both H3.7 and the 15 kDa H3 variants became modified by the homologous PTMs H3.7K3me3 or H3K4me3. Remarkably, unlike H3.3, we did not observe that H3.7 was influenced through Piwi RNAi.

Based on the sequence homology it seems more likely that the acetylation site detected with the anti-H3K36ac pAbs could be LVK105KLPFQ (score 0.69) rather than QSK77KKMKR (score 0.38). Lysine-105 lies in front of the α1 helix of H3, and should be exposed at the lateral surface of the nucleosome, with direct contact to the DNA. Trimethylation of the homologous H3K64 in mammals was associated with the establishment of heterochromatin structure [31]. Therefore, it is possible that H3K105ac could counteract heterochromatin formation at MDSs.

### Piwi knock-down downregulates H3.3 at both the transcript and protein levels

An open problem results from our finding that not only the deposition of the variant histone H3.3 is affected by Piwi knock-down, but also the expression of its gene *HIS33*. Therefore, not only would a mechanistic link between Piwi and the machinery for the selective deposition of H3 variant-containing nucleosomes into chromatin be required, but also a feedback loop for the regulation of histone variant gene expression. The easiest, but improbable, explanation is that Piwi acts as a transcription factor for H3.3. We believe that this hypothesis can be rejected, as H3.3 is permanently expressed during the *Stylonychia* life cycle, whereas the occurrence of Piwi is restricted to a narrow period. It seems rather more likely that Piwi regulates the expression of H3.3 via interaction with H3.3-specific histone chaperones. It was described that in budding yeast, histone chaperones, such as HIR or Asf1, can act as positive or negative regulators of histone genes, depending on their assembly into different complexes during the cell cycle, such as the ATP-dependent chromatin remodeling complexes SWI/SNF or RSC, responsible for either activation or repression of histone genes, respectively [32]. It seems reasonable to assume that for macronuclear differentiation in *Stylonychia,* an active complex containing Piwi, MDS-specific RNAs, histone chaperones, H3.3, and possibly chromatin remodelers could beget a positive feedback loop on H3.3 expression, whereas abolition of this complex via Piwi RNAi would suppress *HIS33*.

## Conclusions

Taken together, our results show that differential H3 variant deposition into nucleosomal arrays correlates with functional chromatin structure discrimination in developing macronuclei during sexual reproduction in *Stylonychia*, thus possibly contributing to determine the fate of specific sequences. Specific variants were selectively targeted by PTM. H3.7 is a development-specific H3 variant that,in its specifically acetylated form,is enriched within sequences that do not undergo programmed DNA elimination. Intriguingly, the deposition of H3.3 during macronuclear differentiation apparently depends on a Piwi-ncRNA pathway. Thus, it is possible that there is a functional connection between this pathway and the assembly of histones into chromatin, but further studies are needed to evaluate this speculative hypothesis.

## Methods

### Adaptation to the new nomenclature for histone variants

Histone variants were partly renamed with respect to a phylogeny-based nomenclature as recently proposed (Table 2) [33].

**Table 2:** New histone variant nomenclature

**Old**	**New**	**Note/GenBank accession**
H3v1	H3.5	KJ159079
H3v2/7/9	H3.2	Possibly three paralogs encoding two protein variants: H3.2a/b; KJ159075, KJ159076
H3v3	H3.1	KJ159074
H3v4	H3.4	KJ159078
H3v5	H3.3	Based on phylogenetic position with regard to animal H3.3; KJ159077
H3v6	Does not exist	Not applicable
H3v8	H3.6	KJ159080
H3v10	H3.7	KJ159081
mdp64	H3.8	Putative protein X; KJ159082

### Growth of *Stylonychia*

Growth of *Stylonychia* and isolation of macronuclei, micronuclei, or macronuclear anlagen were performed as described previously [18].

### RNA interference

For Piwi knock-down during macronuclear development, we cloned a 1040 bp amplicon from the macronuclear PIWI CDS or a mock sequence into the L4440 (double T7) vector. Alternatively, a 222 bp amplicon from the HIS37 CDS was cloned into L4440. Subsequently, this construct was transfected into RNase III-deficient DE3 *Escherichia coli*. These vectors were used for Piwi inhibition or as control, respectively. Briefly, bacteria were added to ciliate cultures 1 to 2 hours prior to algae feeding. Cells were fed for 4 days with bacteria, which expressed double-stranded RNA homologous to Piwi mRNA, similarly to earlier descriptions [34]. Thereafter, conjugation was induced, and the RNAi effects were analyzed from subsequent developmental stages.

### Purification of nucleic acids and cDNA synthesis

DNA and RNA isolation and cDNA synthesis were performed as described previously [16,35].

### Telomere-suppression PCR

*Stylonychia* macronuclear nanochromosomes encoding H3 variants were fully sequenced using degenerate oligonucleotides in combination with telomere-suppression PCR [27].

### Gene expression analyses

Accumulation of mRNAs was analyzed by qPCR on a Rotor Gene 6000 (Corbett Life Science, Hilden, Germany) using QuantiTect SYBR Green Master Mix (Qiagen, Hilden, Germany). For gene expression assessments of histone H3 variant genes, all raw values were normalized against two reference genes (*ACT1* and *αTEBP*), using the geometric mean of at least five repeated measurements. The primers used are listed in Table 3. PCR conditions were as follows: 95°C for 15 minutes, followed by 40 cycles of 95°C for 15 seconds and 60°C for 30 seconds. Melting of PCR product was performed using a temperature gradient from 55°C to 95°C, rising in 0.5°C increments. To calculate relative changes in H3 variants mRNA levels over the life cycle of *Stylonychia*, we applied the ^ΔΔ^Ct method.

**Table 3:** Primers used in this study

**Primer**	** Sequence 5′→3′**
ACT1-	TTGCTGGCGAAGGTTGAGAG
ACT1+	TGCCAGCCCAGACAGAAGAT
AP1	GTAATACGACTCACTATAGGGC
AP12	GTAATACGACTCACTATAGGGCACGCGTGGTCGACGGCCCGGGCT
AP2	ACTATAGGGCACGCGTGGT
H3.1-	GTTGCATGTCCTTTGGCATGATGGTAAC
H3.1+	CACCGGTGGAGTCAAGAAAC
H3.2-	GAGAGCAAGGACAGCTGATGA
H3.2+	GGTACCGTTGCTCTCAGAGAAA
H3.3-	CCTCTGATTCTTCTGGCTAGCTATATATCC
H3.3+	GTCACACTTCACATTTTGTCTC
H3.4-	CAAGTTGAATGTCCTTAGGCATGATTG
H3.4+	CCTGCTGATGGTGGAGTT
H3.5-	GGGCGAGGACAGCAGATGAT
H3.5+	GAAGATTCCAAAAGAGCACTGAACT
H3.6-	GCTAATTGCATATCTTTCGGCATGATG
H3.6+	CCTGCTAATGCCGGAATA
H3.7-	AAAGTGCCAGTAGGCCTTGAATACTG
H3.7+	AAGAAACTCCCTTTCCAGAGATTAG
H3.8-	TGGCGTGAATGGCGCACAA
H3.8+	CTCAAGGCTCCCTTCCAGAGATT
H4–	AAATAGTTGGTTAAAGTTCTCATTC
H4+	ATGATGTGATGTTTTTTTAATGATC
MaA81-	TGTGCCAAGAAGGCCAAAGAG
MaA81+	TCAGTATCACCATCTACAACATTCG
Mdp2-	AGAAGAGGAGGACCGAGTGG
MDP2a-	AAGGAGAGTATAATATGATTGGACTGTTG
MDP2a+	TGCTTGACTGAGTCGTCAGAAT
Mdp2b+	ATCAGTCTCTGAGGGAAATAGGC
pLJ01-	TCAAAGAGGTCGATGGTTCC
pLJ01+	ACCTCGATACCGCATACCAG
Stad5-	AAACATTCACCCCCAAAGC
Stad5+	CATCAAGGACCGCTATTCCTAC

### Antibodies targeted to histone H3 variants

Using peptides, pAbs targeted against three histone H3 variants were raised (BioGenes, Berlin, Germany): guinea pig anti-H3.3 (EQLANKAARKTAQVAQS), rabbit anti-H3.5 (QLANKAARKSTNVNAVS), and rat anti-H3.7 (PANQSKKKMKRFKPG). Use of anti-H3.3 and anti-H3.5 pAbs in Western blots revealed a band of approximately 15 kDa, but different temporal enrichment was seen using chromatin purified from the different nuclear types at different developmental stages. Anti-H3.7 reacted with an approximately 20 kDa band (Figure 2B). Peptide competition assays using the immunizing peptides for competitive blocking of the corresponding antibodies resulted in a loss of signal in Western blot analyses, whereas the use of H3.3 peptide in combination with the H3.5 pAb did not impair H3.5 reactivity or *vice versa*. None of the antibodies reacted with calf thymus histones in Western blot analyses (data not shown). The other antibodies used in this study had been tested previously [16].

### Gel separation and Western blot analyses

Nuclear proteins were resuspended in loading buffer, heated for 10 minutes at 95°C, and separated by SDS-PAGE (15% gels). Proteins were then transferred onto a nylon membrane and probed with specific antibodies. Secondary detection was performed using HRP-conjugated pAbs and enhanced chemoluminescence (ECL) substrate (Pierce/Thermo-Fisher, Rockford, Illinois, USA).

### Chromatin purification, chromatin immunoprecipitation, and quantitative real-time PCR

Chromatin was isolated from polytene macronuclear anlagen (a3). Anlagen nuclei were fixed in PBS with 1% formaldehyde for 10 minutes at room temperature. They were then washed with PBS, and subsequently incubated with glycine stop solution, followed by additional washing with PBS. Nuclei were then resuspended in ice-cold nuclei lysis buffer (50 mM Tris–HCl pH 8.0, 10 mM EDTA, 0.1 mM phenylmethanesulfonylfluoride (PMSF) 1% SDS). Following centrifugation for 10 minutes at 16,100 ×*g* in a microcentrifuge at 4°C, the supernatant containing the soluble chromatin fraction was transferred into a new tube. The chromatin concentration was measured at 260 nm using a NanoPhotometer (Implen, Munich, Germany).

Portions of 50 μg (0.1 ng/μL) chromatin were sheared by ultrasonic treatment using a Bioruptor UCD-200 (Diagenode, Liege, Belgium) and 25 cycles (30 seconds on/30 seconds off) at the ‘high’ position. Chromatin fragment size was assessed by separation in an agarose gel, and one of the chromatin aliquots was saved as input.

For ChIP, 50 μg sheared chromatin were incubated with antibodies targeted to H3K36ac (Millipore, Billerica, Massachusetts, USA) in a rotator for 16 hours at 4°C in a total volume of 250 μl diluted with ChIP incubation buffer (50 mM NaCl, 50 mM Tris–HCl, pH7.5, 0.1 mM PMSF, 5 mM EDTA, and 0.1% SDS). Subsequently, 25 μl of protein G magnetic beads (Active Motif) were added and incubated for 4 hours at 4°C with rotation. The protein G magnetic beads were separated on a magnetic rack and washed repeatedly. To elute enriched DNA fragments, immunocomplexes were incubated with elution buffer (1% SDS, 10 mM EDTA, and 50 mM Tris–HCl pH 8.1) for 30 minutes at 65°C on a shaker. Eluates were treated with proteinase K. DNA was purified using phenol-chloroform extraction and ethanol precipitation.

We performed qPCR analyses as described above. The relative amounts of immunoprecipitated DNA were analyzed in triplicate. The pulled-down DNA fragments were measured as the percentage of input, determined by the ^ΔΔ^Ct method. The primer pairs used are described in Table 3.

### Confocal laser scanning microscopy

Sample treatment for immunofluorescence confocal laser scanning microscopy was performed using the protocol, antibodies, and dyes described in detail previously [16]. Images were assembled using ImageJ (Rasband, W.S., ImageJ, National Institutes of Health, Bethesda, Maryland, USA; http://rsb.info.nih.gov/ij/, 1997–2004) and Adobe Photoshop CS5 software.

## Abbreviations

ACT1: Actin I; Asf1: Anti-silencing function protein 1; CAF-1: Chromatin assembly factor 1; cDNA: Copy DNA; CDS: Coding sequence; ChIP: Chromatin immunoprecipitation; DNA: Deoxyribonucleic acid; HIR: Histone regulatory; HIRA: Histone cell cycle regulation defective homolog A; HRP: Horseradish peroxidase; IES: Internal eliminated sequences; MDP: Macronucleus development protein; MDS: Macronucleus-destined sequence; ncRNA: Non-coding RNA; pAb: Polyclonal antibody; Piwi: P-element Induced Wimpy Testis; PTM: Post-translational modification; PVDF: polyvinylidene fluoride; qPCR: Quantitative PCR; RNAi: RNA interference; RSC: Remodels the Structure of Chromatin; SWI/SNF: SWItch/Sucrose Non-Fermentable; TEBP: Telomere end-binding protein.

## Competing interests

The authors have nothing to disclose.

## Authors’ contributions

SF gathered most of the sequence data, and conducted the Western blot analyses and ChIP experiments. SF and AB performed cloning and RNAi experiments. SF and JP conducted analyses of gene expression. FJ took part in the sequence analyses and helped to write the manuscript. JP designed peptides for antibody production and performed the microscopical analyses. All authors took part in data analysis. HJL and JP designed and supervised the study and wrote the paper. All authors read and approved the final manuscript.

## Supplementary Material

Additional file 1 **Sexual reproduction and replication events in *Stylonychia.*** The illustration shows the sexual cycle of *Stylonychia*. **(A)** Morphological changes and spatiotemporal localization of H3 variants. DNA replication events are highlighted (®). (A_inset_) is a microscopic visualization of nuclear exchange (stage 3), in which cells belonging to one strain were labeled over 48 hours using BrdU. BrdU incorporated into micronuclear and macronuclear DNA (green) was then detected using mouse anti-BrdU mAbs (Sigma Aldrich, St. Louis, Missouri, USA) and anti-mouse-Alexa-Fluor 488 secondary antibodies (Invitrogen, Carlsbad, Califoria, USA). DNA in all cells was counterstained using To-Pro-3 (red). **(B)** DNA content scheme during macronuclear development. The occurrence of nuclear developmental stages (m, a1 to a3, e, M) and timing of important molecular events are indicated using the same abbreviations as in **(A)**. Click here for file

Additional file 2 **Nanochromosomes encoding histone H3 variants. (A)** Alignment of nine full-length nanochromosomes encoding eight histone H3 variants. Telomeres, putative TATA boxes, start and stop codons, a GT-AG type intron, and putative transcription factor binding sites are highlighted. **(B)** Phylogenetic relationship of *Stylonychia* H3 variant protein sequences (neighbor-joining method). *Hydra* H3 was used to root the tree. **(C)** Phylogenetic relationship of *Stylonychia* H3 variant DNA sequences (maximum likelihood method). *Hydra* H3 was used to root the tree. Click here for file

Additional file 3 **H3 variants in *Stylonychia*. (A)** Protein sequence alignment of *Stylonychia* H3 variants and *Hydra* H3.3. Residues conserved in most variants are shaded. Further designated conserved motifs adjacent to prominent post-translational modification (PTM) target sites are highlighted in green (around H3K4), red/orange (around H3K9/K27), blue (around H3K36/K64), or yellow (chaperone recognition domain). Alignments were produced using MEGA5 [36] and manually refined. **(B)** Evolutionary relationships of chaperone recognition sites in *Stylonychia* H3 variants. The evolutionary history was inferred using the maximum likelihood method. Evolutionary analyses were conducted in MEGA5. Click here for file
