# Supplementary material for: Differential expression of histone H3 genes and selective association of the variant H3.7 with a specific sequence class in Stylonychia macronuclear development
Source: Epigenetics Chromatin. 2014 Feb 7;7:4. doi: 10.1186/1756-8935-7-4 (PMC3918171; doi:10.1186/1756-8935-7-4)

Supplemental Material 1

Sexual reproduction and replication events in *Stylonychia*

The illustration shows the sexual cycle of *Stylonychia*. **A.** Morphological changes and spatiotemporal localization of H3 variants. DNA replication events are highlighted (®). A_{inset} is a microscopic visualization of nuclear exchange (stage 3), where cells belonging to one strain were priorly labeled using BrdU over 48h. BrdU incorporated into micronuclear and macronuclear DNA (green) was detected using mouse anti-BrdU mAbs (Sigma) and anti-mouse-Alexa-Fluor 488 secondary antibodies (Invitrogen). DNA in all cells was counterstained using To-Pro-3 (red). **B.** Below is a DNA content scheme during macronuclear development. The occurence of nuclear developmental stages (m, a1-3, e, M) and timing of important molecular events are indicated using the same abbreviations as in A.

A.

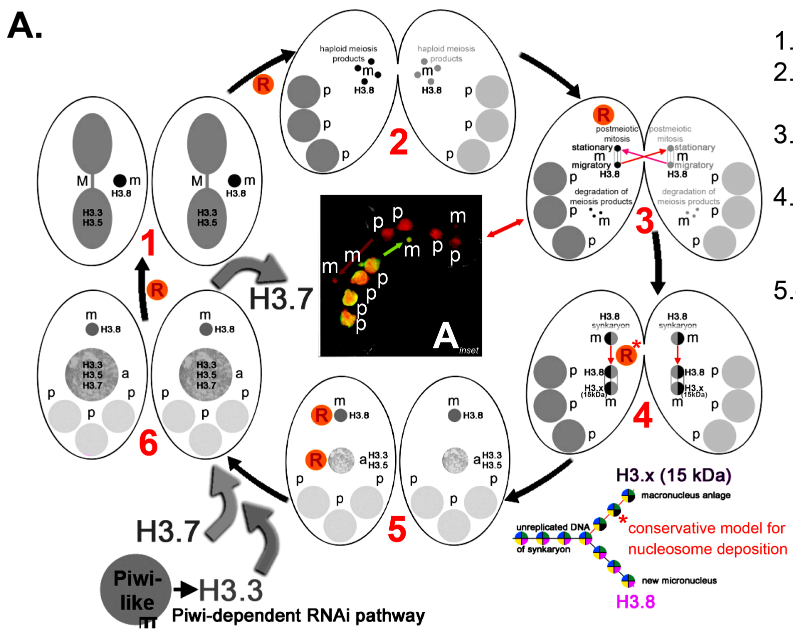

1. Vegetative *Stylonychia*
2. Conjugation: meiosis (micronuclei), fragmentation (parental macronuclei)
3. Conjugation: exchange of haploid micronuclei
4. Conjugation: synkaryon formation, mitosis nucleates new micronucleus and new macronucleus (anlage)

5.-6. Maturation of new macronucleus: polytenization, programmed chromatin reorganization/DNA elimination, telomere addition, nanochromosome replication

m = micronucleus (derivative)

M = macronucleus

p = parental (old) macronucleus

a(1-3) = anlage (developing macronucleus)

R = replication event

B.

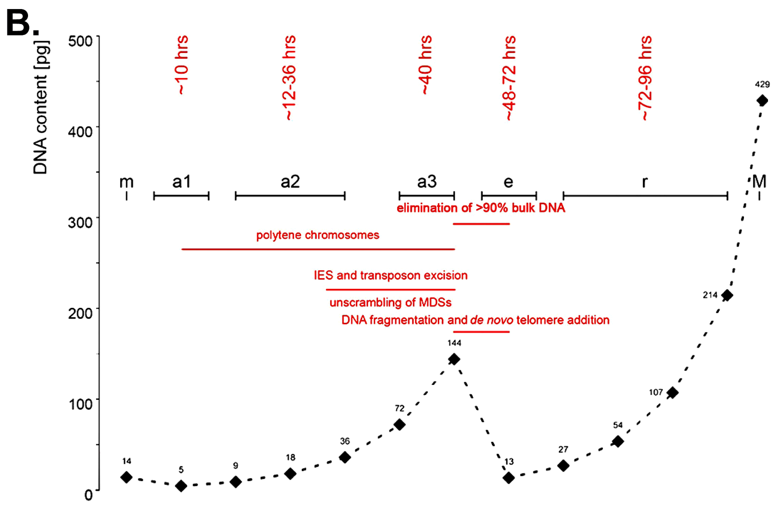

Supplement: Additional file 1 — Sexual reproduction and replication events in Stylonychia. The illustration shows the sexual cycle of Stylonychia. (A) Morphological changes and spatiotemporal localization of H3 variants. DNA replication events are highlighted (®). (Ainset) is a microscopic visualization of nuclear exchange (stage 3), in which cells belonging to one strain were labeled over 48 hours using BrdU. BrdU incorporated into micronuclear and macronuclear DNA (green) was then detected using mouse anti-BrdU mAbs (Sigma Aldrich, St. Louis, Missouri, USA) and anti-mouse-Alexa-Fluor 488 secondary antibodies (Invitrogen, Carlsbad, Califoria, USA). DNA in all cells was counterstained using To-Pro-3 (red). (B) DNA content scheme during macronuclear development. The occurrence of nuclear developmental stages (m, a1 to a3, e, M) and timing of important molecular events are indicated using the same abbreviations as in (A). [file 1756-8935-7-4-S1.pdf]
