# Supplementary material for: Differential expression of histone H3 genes and selective association of the variant H3.7 with a specific sequence class in Stylonychia macronuclear development
Source: Epigenetics Chromatin. 2014 Feb 7;7:4. doi: 10.1186/1756-8935-7-4 (PMC3918171; doi:10.1186/1756-8935-7-4)
Supplement: Additional file 2 — Nanochromosomes encoding histone H3 variants. (A) Alignment of nine full-length nanochromosomes encoding eight histone H3 variants. Telomeres, putative TATA boxes, start and stop codons, a GT-AG type intron, and putative transcription factor binding sites are highlighted. (B) Phylogenetic relationship of Stylonychia H3 variant protein sequences (neighbor-joining method). Hydra H3 was used to root the tree. (C) Phylogenetic relationship of Stylonychia H3 variant DNA sequences (maximum likelihood method). Hydra H3 was used to root the tree. [file 1756-8935-7-4-S2.pdf]

Supplemental Material 2

Nanochromosomes encoding histone H3 variants

A. Alignment of 9 full-length nanochromosomes encoding 8 histone H3 variants. Telomeres, putative TATA boxes, START codons, STOP codons, a GT-AG type intron and putative transcription factor binding sites are highlighted.

B. Phylogenetic relationship of *Stylonychia* H3 variant protein sequences (Neighbour joining method). *Hydra* H3 was used to root the tree.

C. Phylogenetic relationship of *Stylonychia* H3 variant DNA sequences (Maximum likelihood method). *Hydra* H3 was used to root the tree.

A.

```
                 ------Telomere------
HIS31       1    CCCCAAAACCCCAAAACCCCAGGAAACTTTGATAGGATTGAGGAAGTAAAATATAATGTTATAAAATGATAATAATT--T
HIS32A      1    CCCCAAAACCCCAAAACCCC------------------ACCGCAAGAGGAATA-ATTATTATAAAA-GCTAGTTGTTAAT
HIS32B      1    CCCCAAAACCCCAAAACCCC--------------------AGCAAGAGGAATA-ATTATTATAAAA-GCTAGTTGTTAAT
HIS33       1    CCCCAAAACCCCAAAACCCC--------------------------AAAATCCGATAACCCTAATTAAAAAACCGACTTT
HIS34       1    CCCCAAAACCCCAAAACCCC----------------------GTTGGGCGATATTTATGGGTAAATAAGTTTGTATA-TA
HIS35       1    CCCCAAAACCCCAAAACCCC---------------------TCTGTTTAATTAGACTAAATTAA-TCAATAATTCTA--A
HIS36       1    CCCCAAAACCCCAAAACCCC---------------------ATTAATCTGAAATATAGGAC-ATTTTGATAATTGAATTA
HIS37       1    CCCCAAAACCCCAAAACCCC-------------------AGAGTGTTGAAATATCTTATTCAATCATAATTAAAGCATAA
HIS38       1    CCCCAAAACCCCAAAACCCC---------------------ATTGTACAAATATTTAACAAAAATTCAGTTGTTGGAATA

                                                                                  putative NF-Y site
HIS31      79    TTAACATTCAATATGGAAAAAG---ATGCAAGGCTGAGTGATTGGTTTCTCACTTAAGC-CTGATCTTCTGATTGGTTCA
HIS32A     61    TTAAAATTTAATGTCAAAAAGGCATGTTTCATCTTTGATGATTTCAATTTAAACAAGCA-CTTAGCTTATAAAAATATTA
HIS32B     59    ATAAAATTTAATGTCAAAAAGGCATGTTTCATCTTTGATGATTTCAATTTAAACAAGCA-CTTAGCTTATAAAAATATTA
HIS33      55    TCACTGCAGAAGAATGAT--TTATATTTAGAAGTTTAAGTGATAAGAGGATTTGTGCCTACAAATTAATGCCTTGTGCCC
HIS34      58    TTTCTAAAAAAAAATATTAATAA---TTTGATTTTAATAATACAAAGATTGACAAGCAT-CTAACAATTAGGATATTTAA
HIS35      57    TATTTAGAATACCGCTTTAATTCTTCTTGAATATTGGTCTGACTGC-CCTATTTAAGGC-AGCCAGACTCAATTAATTAT
HIS36      59    TTTA-ATTTTTATTAATCAGTTA---GTATATACAAGTGCAATTTTAATTAAATCTGTTAAAAGGAATTGATATGCACTT
HIS37      62    TAACCAATCAAATCTAAC--TCA---CTGAAATTTAAATTAACTAAATTAAACTCA-AT-CAAATTATCAGTATAAAAGG
HIS38      60    TAAATAGCAGAATT-AATAATTAATTTTCAAATTCAAATTTTA--AACAAACTC----TCAAGA----------------

HIS31     155    ---TTTAATTATATTTTCGAGAAGGGGTTTAATTTTATCTAC-CATTAAATATT-TGAAAGTGA--AAGTCATATATATG
HIS32A    140    GGAACCAAATGAATATATTAGTTATGTTTGAATCTAATAGACTCATGTCTTATTATGAACATGAATAAACCTAATTAGTC
HIS32B    138    GGAACCAAATGAATATATTAGTTATGTTTGAATCTAATAGACTCATGTCTTATTATGAACATGAATAAACCTAATTAGTC
HIS33     133    TAGTCTCTGAGCTAGAGTTATTAAATAGGATCCACCAAGACCGATTTATTCTATAGTAATAAATTTACAGACTACTAAAC
HIS34     134    AGCTAGCTCGGTCAACAAGAACTCAAACTTTTCAAAACAATCTATACAGATAAGAA------------------------
HIS35     135    AATTAAATTTAAAAATTTTACAACAACACCTAACCAACAAGAG-------------------------------------
HIS36     135    AAATCCCCTAGAATCCATTATTAATCAAGATAATCCGAATAATTAGATATTTAAGTCAGGCTTAACAAGGATACTATAAT
HIS37     135    AAGTTTAAAAGTCAAAATTATAATTAATTGTTTGACA-------------------------------------------
HIS38     118    --------------------------------------------------------------------------------

HIS31     228    TATTCATGGAGCATTGTCCTCTGAGGTTAGAATGTGTTTAATTAAT-TAATTAATATCA-GTGTAATCTTCAAACTTAAT
HIS32A    220    AAGATGTTTAGGAGAAATGACATATTTTTTAACTTGATTAATTAATCTAGTCATTGCTATAGGCAACCTCATATTTTGAT
HIS32B    218    AAGATGTTTAGGAGAAATGACATATTTTTTAACTTGATTAATTAATCTAGTCATTGCTATAGGCAACCTCATATTTTGAT
HIS33     213    AAACCT--------------------------------------------------------------------------
HIS34     190    --------------------------------------------------------------------------------
HIS35     177    --------------------------------------------------------------------------------
HIS36     215    AAAAACAAATCCAAACCTCATCATCTCCTTATTAGCA-------------------------------------------
HIS37     172    --------------------------------------------------------------------------------
HIS38     118    --------------------------------------------------------------------------------

HIS31     306    CTGAGTTTGTTTCTATTATAATATAA-TTTAATAATGCTGTAAAATTGATAAGATCAGTACAATTGCCAACAATATTTAG
HIS32A    300    TTATTTATATATTTTATACAAAAAAAATGAAAAAATGTACCTGAAATACTAGTCTTCTCCTATAAAGTATCGATATAATT
HIS32B    298    TTATTTATATATTTTATACAAAAAAA-TGAAAAAATGTACCTGAAATACTAGTCTTCTCCTATAAAGTATCGATATAATT
HIS33     219    --------------------------------------------------------------------------------
HIS34     190    --------------------------------------------------------------------------------
HIS35     177    --------------------------------------------------------------------------------
HIS36     252    --------------------------------------------------------------------------------
HIS37     172    --------------------------------------------------------------------------------
HIS38     118    --------------------------------------------------------------------------------
```

```
HIS31    385 AATTAGAGATTTCTATGGCAA--TATCGGCCGAAATTCATGAGAAATATTTATTGATCAAAATGCTTGATTAGGAGCCAT
HIS32A   380 CTTTAAAAATAACTCTGGAAAAATATCAATTGACACAAAGATGACATAATGATTCATTTGTATAAATCATCTTCATGAAT
HIS32B   377 CTTTAAAAATAACTCTGGAAAAATATCAATTGACACAAAGATGACATAATGATTCATTTGTATAAATCATCTTCATGAAT
HIS33    219 --------------------------------------------------------------------------------
HIS34    190 --------------------------------------------------------------------------------
HIS35    177 --------------------------------------------------------------------------------
HIS36    252 --------------------------------------------------------------------------------
HIS37    172 --------------------------------------------------------------------------------
HIS38    118 --------------------------------------------------------------------------------

HIS31    463 TATCTCAAGCATTTCTTTGCTTAA--TTAAGGACCATGACAATGACAATTAATGGA--AATAACAGAGAAATTGTGATGG
HIS32A   460 TTACCAAGAAATGCTTAAACTTGAGACTCATGAATTCCTAGATGACTCTCTATTGATAAATGATTAAATGACGGGGTAAC
HIS32B   457 TTACCAAGAAATGCTTAAACTTGAGACTCTTGAATTCCTAGATGACTCTCTATTGATAAATGATTAAATGACGGGGTAAC
HIS33    219 --------------------------------------------------------------------------------
HIS34    190 --------------------------------------------------------------------------------
HIS35    177 --------------------------------------------------------------------------------
HIS36    252 --------------------------------------------------------------------------------
HIS37    172 --------------------------------------------------------------------------------
HIS38    118 --------------------------------------------------------------------------------

HIS31    539 ATTGACTAGCATGT-CTCTGTTTGCTCGAATAATAATTGGAAAATAGTAAAACTATTGGAAGAATTATGAGTAA-ATCCT
HIS32A   540 TTTGCATAAAATCCACTCTATTTAACCAAA--GTTTTCAAAAAGTAGTGG--TTTACGAGGTTTTTGTGAAAATTATCCA
HIS32B   537 TTTGCATAAAATCCACTCTATTTAACCAAA--GTTTTCAAAAAGTAGTGG--TTTACGAGGTTTTTGTGAAAATTATCCA
HIS33    219 --------------------------------------------------------------------------------
HIS34    190 --------------------------------------------------------------------------------
HIS35    177 --------------------------------------------------------------------------------
HIS36    252 --------------------------------------------------------------------------------
HIS37    172 --------------------------------------------------------------------------------
HIS38    118 --------------------------------------------------------------------------------

                           putative AP-1 site
HIS31    617 AGGTGTATGAACAGATATAGCATATTAGCCAATAAGAGCCAATAATTTGGAAGATAGATAAAGCGAAGCATAATAAATTT
HIS32A   616 AGATAGGCAAGGATAAATGAGTCAATAGTCGCAATCAACCAATCAAGTAAGAGATAGAGATATGAAAAGGGTATAAATTC
HIS32B   613 AGATAGGCAAGGATAAACGAGTCAATAGTCGCAATCAACCAATCAAGTAAGAGATAGAGATATGAAAAGGGTATAAATTC
HIS33    219 --------------------------------------------------------------------------------
HIS34    190 --------------------------------------------------------------------------------
HIS35    177 --------------------------------------------------------------------------------
HIS36    252 --------------------------------------------------------------------------------
HIS37    172 --------------------------------------------------------------------------------
HIS38    118 --------------------------------------------------------------------------------

                                                                      START
HIS31    697 GAATTCAATTAAGACTC--TATAAATTCTATGTTTCAAACA--CAAACTATATAAA-ATGGCAAGAACCAAGCAAACC--
HIS32A   696 AAATTC--TTAAGTAGAACTATAATTCATATTCTAAACAACTCAACAAAACAATAAAATGGCAAGAACCAAGCAAACC--
HIS32B   693 AAATTC--TTAAGTAGAACTATAATTCATATTCTAAACAACTCAACAAAACAATAAAATGGCAAGAACCAAGCAAACC--
HIS33    219 ---------------------------------------------------------ATGGCAAGAACAAAGCAAACA--
HIS34    190 ---------------------------------------------------------ATGGCAAGGACAAAATAAACC--
HIS35    177 ---------------------------------------------------------ATGGCAAGAACCAAGTAGACT--
HIS36    252 ---------------------------------------------------------ATGGCCAGAACAAAGTAAACT--
HIS37    172 ---------------------------------------------------------ATG---AGAACTAAGAGTGCAGC
HIS38    118 ---------------------------------------------------------ATGCCCAGACAAAAGCTCGCCGG

HIS31    770 --------------------------------------------------------------------------------
HIS32A   772 --------------------------------------------------------------------------------
HIS32B   769 --------------------------------------------------------------------------------
HIS33    240 --------------------------------------------------------------------------------
HIS34    211 --------------------------------------------------------------------------------
HIS35    199 --------------------------------------------------------------------------------
HIS36    273 --------------------------------------------------------------------------------
HIS37    192 ATCACCCAACTACTCTATACTGAAAGGAAAAGGAAAAGGTAAGGGCAAGGGCAAGCATCTGAACAGAATGCAAGTCACTA
HIS38    141 TAAGAAGATGAAGTCCTCAACCAAGAAGAGTGTTGCCTCAGGCTCCAAG-------------------------------

HIS31    770 --------GCAAGAAAGAACACTGGTGGCAAGGCCCCAAGAAAGCACATTGCTCACAAA---------------CAAGCT
HIS32A   772 --------GCAAGAAAGAACACTGGAGGCAAAGCCCCAAGAAAGCACATTGCACACAAA---------------CAAGCT
HIS32B   769 --------GCAAGAAAGAACACTGGAGGCAAAGCCCCAAGAAAGCACATTGCACACAAA---------------CAAGCT
HIS33    240 --------GCAAGAAAGAACACTGGTGCTAAAGCCCCAAGAAAATAACTGGCAAACAAA---------------GCTGCA
HIS34    211 --------GCTAGAAAATAAACTGGTGCAAAGGCTCCAAGAAAGGCCCTAGCTAATAAA---------------GCTGCA
HIS35    199 --------GCTAGAAAGAACACTGGTGCTAAAGCTCCAAGAGAGCAACTCGCTAACAAG---------------GCCGCA
HIS36    273 --------GCAAGAAAAAATACTGGAGCTAAAGCCCCAAGAAAGCATTTAGCAAATAAA---------------GCCGCA
HIS37    272 GTCAAGAGGCTGTCAATTCTACTACAGATGTCATGAGCTTCGGGAAGATTTCTAATGTTGAGATGCCAGCTCAAGCAGCC
HIS38    190 --------GCCAGCAAGTCAACCATG---------------------------------------------------AAA
```

```
HIS31   827 AAGAAATCATCAGCTGCCGCCGCC---------------ACCGGTGGAGTCAAGAAACCA---CACAGATTCAGACCAGG
HIS32A  829 AAGAAATCATCAGCAGCAGCTGCT---------------ACTGGTGGTGTCAAGAAGCCA---CACAGATTCAGACCAGG
HIS32B  826 AAGAAATTATCAGCAGCAGCTGCT---------------ACTGGTGGTGTCAAGAAGCCA---CACAGATTCAGACCAGG
HIS33   297 AGAAAGAGCACTAATGTAAATGCTGTC------------AGT---GGTGTCAAGAAGCCC---CATAGATTCAGACCTGG
HIS34   268 AGAAAGACTGCT------CCTGCT---------------GATGGTGGAGTTAAGAAACCT---CACAGATTCAGACCAGG
HIS35   256 AGGAAGACTGCCTAAGTAGCCTAG---------------AGTGGCGGAGTCAAGAAGCCA---CACAGATTCAGACCAGG
HIS36   330 AGAAAGACAGCTGCT---CCTGCT---------------AATGCCGGAATAAAAAAGCCT---CATAGATACAGGCCAGG
HIS37   352 AGAAAAATGGCTAAACTTCCAGTCACCACTGTTGCTCCAGCTAACTAATCCAAGAAGAAGATGAAGAGATTCAAGCCTGG
HIS38   211 AAGAAGACT------GCTCCAGCT---------------GAGGGAGGTATCAAGACCCACAACAGAAGATTCAAGCCAGG

                                                                                GT-AG intron
HIS31   889 TACCGTCGCTCTCAGAGAAATCAGAAGATTCCAAAAGAGC----------------------------------------
HIS32A  891 TACCGTTGCTCTCAGAGAAATCAGAAGATTCCAAAAGAGC----------------------------------------
HIS32B  888 TACCGTTGCTCTCAGAGAAATCAGAAGATTCCAAAAGAGC----------------------------------------
HIS33   359 CACTGTCGCATTAAGAGAGATCAGAAGATTCCAAAAATCTGTAAGTCACACTTCACATTTTGTCTCCATCTACCTCATAA
HIS34   324 TACTGTGGCACTCAGAGAAATCAGAAAATATCAGAAGTCA----------------------------------------
HIS35   318 AACCGTCGCCCTCAGAGAAATCAGAAAATTCCAGAAGTCC----------------------------------------
HIS36   389 TACAGTAGCACTAAGAGAAATAAGAAAGTATCAAAAGAGC----------------------------------------
HIS37   432 ATAACTTGCCCTTAAGCAGATTAAGAAACTTCAGTCGAGT----------------------------------------
HIS38   270 TACCGTCGCTCTCAGAGAGATCAAGAGATACTAGAAGGTC----------------------------------------

HIS31   929 ----------------------------ACTGAACTCCTCATCAGAAAGCTCCCCTTCCAAAGACTCGTTAGAGAAATCG
HIS32A  931 ----------------------------ACCGAGCTCCTCATCAGAAAGCTCCCCTTCCAAAGACTCGTCAGAGAAATCG
HIS32B  928 ----------------------------ACCGAGCTCCTCATCAGAAAGCTCCCCTTCCAAAGACTCGTCAGAGAAATCG
HIS33   439 ATTTTCTTTTACTCACGCTCATTTATAGACTGAACTTCTTATCAGAAAATTACCCTTCCAGAGACTAGTAAGGGAAATCG
HIS34   364 ----------------------------ACTGAATTGCTGATCAGAAAGTTGCCATTCTAGAGACTTGTGAGAGAAATTG
HIS35   358 ----------------------------ACAGAGCTTTTGATCAGAAAGCTGCCATTCCAGAGACTTGTGAGAGAAATCG
HIS36   429 ----------------------------ACCGATCTATTAATAAGGAAGTTACCGTTCCAAAGACTCGTCCGTGAGATAG
HIS37   472 ----------------------------ACTGATTTGCTGGTCAAGAAACTCCCTTTCCAGAGATTAGTCAGGGAAATCG
HIS38   310 ----------------------------ACCAACCTCCTCCTCCTCAAGGCTCCCTTCCAGAGATTCGTCAGAGCCATCT

HIS31   981 CCTCAGAGTTCAAGAACGACCTCAGATTCCAATCATCTGCTGTCCTCGCCCCCCAAGAAGCCTCCGAGGCCTACCTCGTC
HIS32A  983 CTTCTGAGTTCAAGAACGACCTAAGATTCCAATCATCAGCTGTCCTTGCTCTCCAAGAAGCCTCAGAGGCTTACCTCGTA
HIS32B  980 CTTCTGAGTTCAAGAACGACCTAAGATTCCAATCATCAGCTGTCCTTGCTCTCCAAGAAGCCTCAGAGGCTTACCTCGTA
HIS33   519 CCTAGGAATACAAAAGTGATCTGAGATTCCAAAGCTAAGCAGTCTTGGCCCTTCAGGAAGCAGCCGAAGCTTACCTAGTC
HIS34   416 CTTCAGATTACAAAAGTGACTTGAGATTCTAATCCTCAGCTGTAGCTGCCATTTAGGAAGCAGCCGAAGCATACATGGTC
HIS35   410 CCTAGGAGTACAAAAGCGATCTCAGATTCCAAAGCCAGGCTGTATTGGCTCTGCAAGAGGCTGCTGAAGCCTACATGGTA
HIS36   481 CATCTGAATACAAAAACGACTTAAGGTTTCAGTCCTCCGCCGTACTTGCACTGTAGGAAGCTGCCGAAGCATACATGATT
HIS37   524 CTCGAAGCAGCAATCCAGAGATCAGATTCAGTATTCAAGGCCTACTGGCACTTTAGGAAGCAGCTGAATGCTTCCTAGTC
HIS38   362 GCGAAGGAATCGACGCCCAACTCAGATTCTAGTCACAAGCCCTCCTCGCCGTCCAAGAAGCTGCTGAGATGTACTTGGTC

HIS31  1061 GGTCTCTTTGAAGACACCAACCTCTGCGCCATCCACGCCAAGAGAGTTACCATCATGCCAAAGGACATGCAACTCGCCAG
HIS32A 1063 GGTCTTTTCGAAGACACCAACCTCGCTGCCATCCACGCCAAGAGAGTCACCATCATGCCAAAGGACATGCAACTCGCTAG
HIS32B 1060 GGTCTTTTCGAAGACACCAACCTCGCTGCCATCCACGCCAAGAGAGTCACCATCATGCCAAAGGACATGCAACTCGCTAG
HIS33   599 GGTTTGTTTGAAGATACCAATCTGTGTGCCATCCATGCTAAGAGAGTGACAATTATGCCTAAGGATATATAGCTAGCCAG
HIS34   496 GGGCTATTTGAAGACACTAACTTGTGTGCCATACATGCTGGAAGAGTCACAATCATGCCTAAGGACATTCAACTTGCTAG
HIS35   490 GGCCTGTTTGAGGACACCAACCTTTGCGCCATTCACGCCAAGAGAGTCACCATTATGCCAAAGGACGTTCAACTCGCCAG
HIS36   561 GGATTATTTGAAGATACAAATCTATGCGCAATACACGGTAAAAGAGTTACCATCATGCCGAAAGATATGCAATTAGCCAG
HIS37   604 GGGCTATTTGAGGACTGCCATCAATGCGCTATCCATGCCAACAGGGTTACGGTTATGCCCAAGGATATGCAGCTAGCTCG
HIS38   442 GGACTTTTCGAAGACTCCAACTTGTGCGCCATTCACGCCAACAGAGTGACTGTCATGAAGAAGGACATGGAGCTCGCCAG

HIS31  1141 AAGAATCAGAGGTGAGAGATCT----------------------------------------------------------
HIS32A 1143 AAGAATCAGAGGTGAGAGATCT----------------------------------------------------------
HIS32B 1140 AAGAATCAGAGGTGAGAGATCT----------------------------------------------------------
HIS33   679 AAGAATCAGAGGAGAAAGAACT----------------------------------------------------------
HIS34   576 AAGAATCAGAGGGGAGAGATCA----------------------------------------------------------
HIS35   570 AAGAATCAGAGGAGAAAGATCA----------------------------------------------------------
HIS36   641 GCGTATTAGGGGTGAGAGATCG----------------------------------------------------------
HIS37   684 CAGACTAAGAGGTGAGAGACAAGATGCC----------------------------------------------------
HIS38   522 AAGAATCAGAGGAGAGAGATTCCACGACCACAGAGACCTCCAACCCAAGAATGGGGATGAAGTTTTCTACCAGTTGCCCT

                                                                 STOP
HIS31  1163 -----------------------------------------------------TGAACATCTCTTCCTTAATGACTTATA
HIS32A 1165 -----------------------------------------------------TGAATCACTAAAGAGCACAAATACCAA
HIS32B 1162 -----------------------------------------------------TGAACCACTAAAGAGCACAAATACCAA
HIS33   701 -----------------------------------------------------TGATACCAGCTCAACTAGTGAGTGATT
HIS34   598 -----------------------------------------------------TGAGGAAGAGATAGCCAAGTGACTCAC
HIS35   592 -----------------------------------------------------TGAGGAGGGTCTGAACATCTAGCATGA
HIS36   663 -----------------------------------------------------TGAAAACTTCTTGTTTCACCGAATCTT
HIS37   712 -----------------------------------------------------TGATGTCACCTCAATGATCGGGTCTTC
HIS38   602 ACTACAACGAGAAGGAGCAGATGGCTCACCTCAAGAAAGTTATCGGCAGAAACTGAACCCGGCAGCATCTCACTTCTCCC
```

```
HIS31  1190 GCAGCAGCGTTATCTATTTTTAATTAGTCTAAATATTAAAAACAATACCTTCACCTATGATATATACTTCTATATTCTAT
HIS32A 1192 TATACAGATATTCAAATTAATCGTGTTATCTATAATTAATTGACTATAAAACCTATATTCACTTACTCTTTATACACATA
HIS32B 1189 TATACAGATATTCAAATTAATCGTGTTATCTATAATTAATTGACTATAAAACCTATATTCACTTACTTTCCATATACATA
HIS33   728 AAAATTTTGATTATCATTAGAACCTAAATATCCCATAATTTTTCTTAAAACAACATCTTTCTTGTCTTTTTGTCATTGTT
HIS34   625 CTTTGGAGTTGAATTAAAGGATAATTAGAGTGATATGATTATTAAGGTAATTATATACTTTTATAAAAATTCGCATAATC
HIS35   619 GGAGAGAGCATGCTAGCAACTATGCTGTTGAGGAGTAATTGATAAGGACTCTGAGAATGAGTACGTTAATTAATTGGTAT
HIS36   690 ACTGATATTTATTTGAAAAATAGTCTCTGTGTAATTACTTGTATAAATTTCCTTAACATTTAATCTCAACCTAATCTCGT
HIS37   739 AATGAGGACTGAGAATTTCATGATCTGCTTGCCTTTCAAGAATTTAATTTCAGTTGCTTGTCAATGAAGCTTACTACAAA
HIS38   682 TAACTCAAAATTAACTATTTG-CTATGGCCATCATATATTTGTATTTTATAATAATTTAGAAAATCCCCTCTTTATAATT

HIS31  1270 TTTATTATTTTACTGCAGAATATTTCTCTTTAAGACTCTAAGTTTCAAAGGACCGCCATTTAAGATTTTAAATAGAGCGG
HIS32A 1272 TATCTATATAAATACTGTTTTTGTCTAAAGTTCCTGAAATATCTCCCACAGAGTTTGGATATTTTTCTAAAATTACTAAG
HIS32B 1269 TATCTATATAAATACTATTTTTGACTAAAATTCCTGAAATATCTCCCACAGAGTTTGGATATTTTTTCCAAATTACTAAG
HIS33   808 CCACTAATGTCTCTTGATTATTTTACCAAATGACCATGAATAAAACTAAT------------------------------
HIS34   705 CAAGCATTCTTCTCAGACTGATTTATTCACTTATAAAATAGGGTAATTTTCAGATTACTAATTAAAATCTTGTATATTAT
HIS35   699 AAAAGGATTTAATATAATATTTGTTGGCAATCTTTCTTTTTTATTTGGTCATTTATTTGATATTCTTCAAACTCATTATT
HIS36   770 GAATTTACAATCATTTGAAGTTCTAGTATCTATCTTTATTAACTTTTAATAGCCTTGGAGTATGATAGGCTTTATGTAAT
HIS37   819 AATACTCTAATCTTTCAGTACTCACTCTACTATGAATTCTCTACATACTACTATACTTTTAACTAGCAGAAATTATAAAC
HIS38   761 ACAATCCTAAATTCAAT-ATAACTTCAAACTCATCATTTTCCTTTCATCTCCCGTGTTTTTTCTTGGGACAAATCTTAAA

HIS31  1350 TTCTTTTTAATGATTTGCTTTATCCGACTACTAAATTAGATAGCCACAATATAATTTTTTATTTTTAATTCTATTGGAAA
HIS32A 1352 TTCCATATAAAAAATGGAGATTTTTCTTTGTTCTGTTAAGAATTCTTCTTATTTTAAACGGCAAAAATATTCTTAATCTT
HIS32B 1349 TCTCCATATAAAAAATGGAGATTTTTCTTTGTTCTGTTAAGAATTCTTCTTATTTTAAACGGCAAAAATATTCTTAATCT
HIS33   858 --------------------------------------------------------------------------------
HIS34   785 TAAATCAACTTTAATTTATTTTTATTTAACTCAATATCGATATCATAATCAATAAA------------------------
HIS35   779 GATATTTATCTAATTTTAATTTCATTATTGAATTTCGAAATATCCTCATCTTCACAAGCTACTTTCCTAAT---------
HIS36   850 AAACGGTGAAGACAAATTTTAAGTAGATCCGAGTAAATTCTGGTACTGAATAATTCTTAAAATAATAAATAAAATAGATA
HIS37   899 CTTATACTTTTAATTTACTTTACTTATTTAAATCTTACTGATTATTTATTATCTTTATTATCTTTTATTTC---------
HIS38   840 GTCTCTTGTGAGTTCGTCAATAATCCTCTAAAAGTCCGTTAAACATATATATTTCTTGCGGATTTATTATACTCTCAGTC

HIS31  1430 TTCCACCATTTACACTCAA-------------------------------------------------------------
HIS32A 1432 TATATATAATAAGGTAAATATAGAGATATCTTTAGACATGCAAAGATCT-------------------------------
HIS32B 1429 TTATATATAATAAGGTAAATATAGAGATATCTTTAGACATGCAAAGATC-------------------------------
HIS33   858 --------------------------------------------------------------------------------
HIS34   841 --------------------------------------------------------------------------------
HIS35   850 --------------------------------------------------------------------------------
HIS36   930 AAAGTAAAAATAAAAGGGCATGTCCGTGCAGGGATTCGAGCCCTGGTTTCTTCGGCCACAACGAAGGGTACTAACCACTA
HIS37   970 --------------------------------------------------------------------------------
HIS38   920 ATATGTGAGATTTATTACTTTTATTAACATTTACACATAATTCATTTACCAAACTTACTTCAAAT---------------

HIS31  1449 --------------------------------------------------------------------------------
HIS32A 1481 --------------------------------------------------------------------------------
HIS32B 1478 --------------------------------------------------------------------------------
HIS33   858 --------------------------------------------------------------------------------
HIS34   841 --------------------------------------------------------------------------------
HIS35   850 --------------------------------------------------------------------------------
HIS36  1010 TACGACACGGACGGGCATATCCTCTATCTAAATTTCTTACATATAAATATCATTTACTATTGAGTGGTAGTCTCACAGTC
HIS37   970 --------------------------------------------------------------------------------
HIS38   985 --------------------------------------------------------------------------------
HIS31  1449 --------------------------------------------------------------------------------
HIS32A 1481 --------------------------------------------------------------------------------
HIS32B 1478 --------------------------------------------------------------------------------
HIS33   858 --------------------------------------------------------------------------------
HIS34   841 --------------------------------------------------------------------------------
HIS35   850 --------------------------------------------------------------------------------
HIS36  1090 AGTTCCTGATTATATACCATTAAAGATATTCATTTGACATGATCTATATATTCTAACTAAATTCTTATCAATCTTGGCCT
HIS37   970 --------------------------------------------------------------------------------
HIS38   985 --------------------------------------------------------------------------------

               --------------Telomere--------------
HIS31  1449 ---GGGGTTTTGGGGTTTTGGGGTTTTGGGGTTTTGGGG
HIS32A 1481 ---GGGGTTTTGGGGTTTTGGGGTTTTGGGGTTTTGGGG
HIS32B 1478 ---GGGGTTTTGGGGTTTTGGGGTTTTGGGGTTTTGGGG
HIS33   858 ---GGGGTTTTGGGGTTTTGGGGTTTTGGGGTTTTGGGG
HIS34   841 ---GGGGTTTTGGGGTTTTGGGGTTTTGGGGTTTTGGGG
HIS35   850 ---GGGGTTTTGGGGTTTTGGGGTTTTGGGGTTTTGGGG
HIS36  1170 ATTGGGGTTTTGGGGTTTTGGGGTTTTGGGGTTTTGGGG
HIS37   970 ---GGGGTTTTGGGGTTTTGGGGTTTTGGGGTTTTGGGG
HIS38   985 ---GGGGTTTTGGGGTTTTGGGGTTTTGGGGTTTTGGGG
```

B.

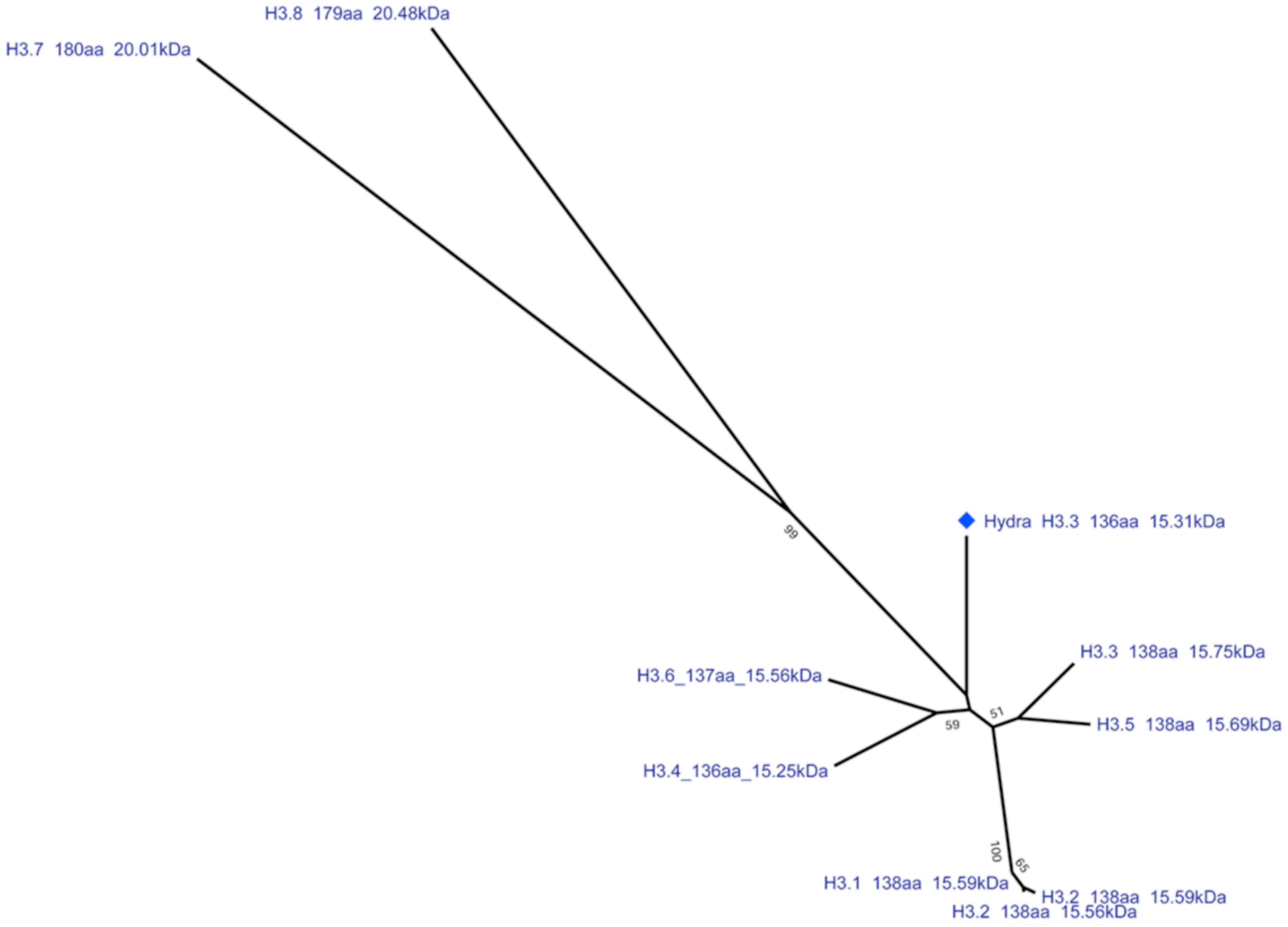
H3.8 179aa 20.48kDa
H3.7 180aa 20.01kDa
99
Hydra H3.3 136aa 15.31kDa
H3.3 138aa 15.75kDa
H3.6_137aa_15.56kDa
51
H3.5 138aa 15.69kDa
59
H3.4_136aa_15.25kDa
100
65
H3.1 138aa 15.59kDa
H3.2 138aa 15.59kDa
H3.2 138aa 15.56kDa

C.

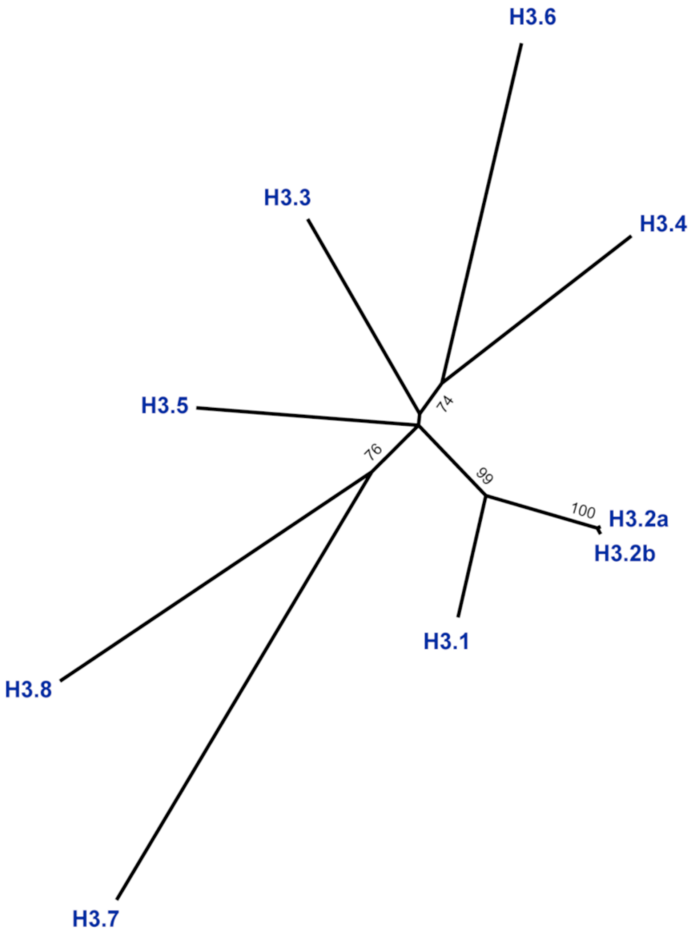
H3.6
H3.3
H3.4
H3.5
74
76
99
100
H3.2a
H3.2b
H3.1
H3.8
H3.7
