# Supplementary material for: Differential expression of histone H3 genes and selective association of the variant H3.7 with a specific sequence class in Stylonychia macronuclear development
Source: Epigenetics Chromatin. 2014 Feb 7;7:4. doi: 10.1186/1756-8935-7-4 (PMC3918171; doi:10.1186/1756-8935-7-4)

Supplemental Material 3: H3 variants in *Stylonychia*

A. Protein sequence alignment of *Stylonychia* histone H3 variants and *Hydra* H3.3. Residues conserved in most variants are shaded, further designated conserved motifs adjacent to prominent PTM target sites are highlighted in green (around H3K4), red/orange (around H3K9/K27), blue (around H3K36/K64) or yellow (chaperone recognition domain). Alignments were done using MEGA5 (36) and manually refined.

B. Evolutionary relationships of the chaperone recognition sites in *Stylonychia* H3 variants. The evolutionary history was inferred using the Maximum likelihood method. Evolutionary analyses were conducted in MEGA5.

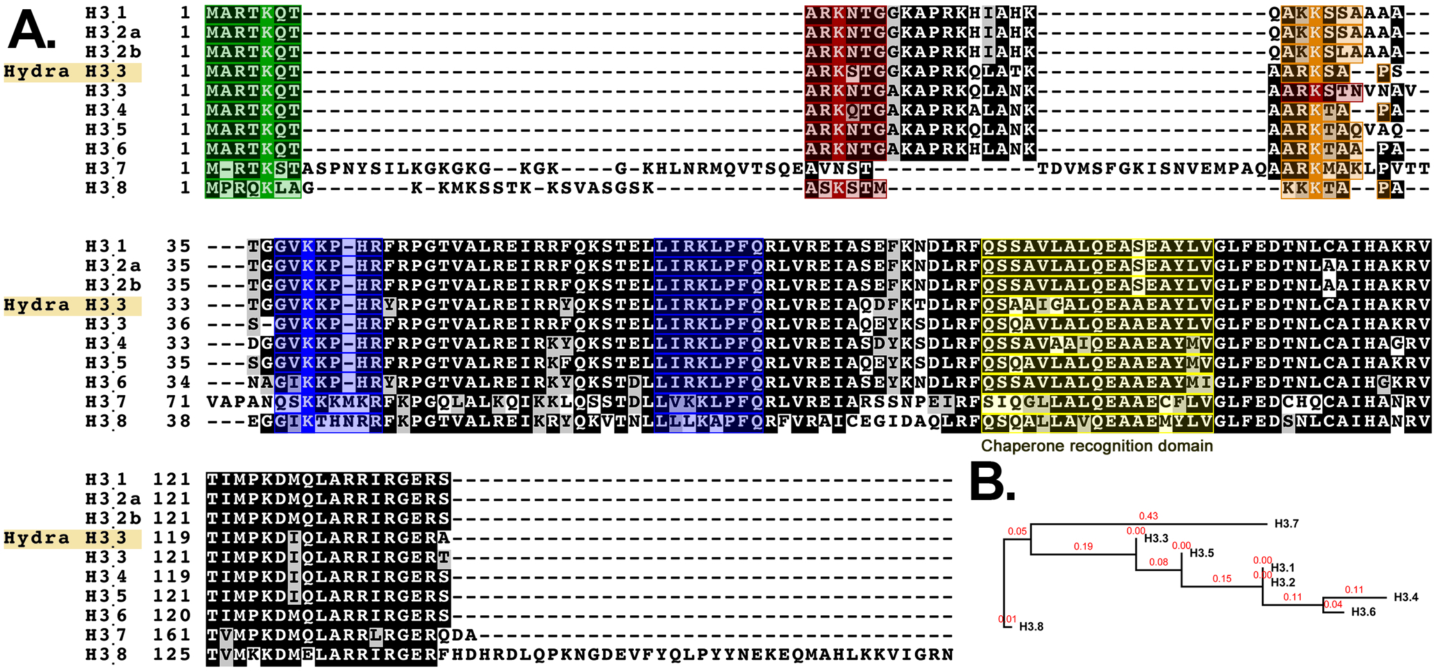

Supplement: Additional file 3 — H3 variants in Stylonychia. (A) Protein sequence alignment of Stylonychia H3 variants and Hydra H3.3. Residues conserved in most variants are shaded. Further designated conserved motifs adjacent to prominent post-translational modification (PTM) target sites are highlighted in green (around H3K4), red/orange (around H3K9/K27), blue (around H3K36/K64), or yellow (chaperone recognition domain). Alignments were produced using MEGA5 [36] and manually refined. (B) Evolutionary relationships of chaperone recognition sites in Stylonychia H3 variants. The evolutionary history was inferred using the maximum likelihood method. Evolutionary analyses were conducted in MEGA5. [file 1756-8935-7-4-S3.pdf]
